# One-pot installation of N-heterocyclic carbenes on gold nanoparticles through counterion-dependent mild-base activation

**DOI:** 10.1039/d6nr01951j

**Published:** 2026-09-02

**Authors:** Robert Richstein, Constantin Eisen, Sophie R. Thomas, Jia Min Chin, Michael R. Reithofer

**Affiliations:** a Institute of Inorganic Chemistry, Faculty of Chemistry, University of Vienna Währinger Str. 42 1090 Vienna Austria michael.reithofer@univie.ac.at; b Institute of Functional Materials and Catalysis, Faculty of Chemistry, University of Vienna Währinger Str. 42 1090 Vienna Austria jiamin.chin@univie.ac.at; c Vienna Doctoral School in Chemistry, University of Vienna Währinger Str. 42 1090 Vienna Austria; d Wolfgang-Pauli-Institute Oskar-Morgenstern-Platz 1 1090 Vienna Austria

## Abstract

N-heterocyclic carbene (NHC)-stabilized gold nanoparticles offer exceptional chemical and thermal stability, yet their installation typically requires multistep procedures, strong bases, or strictly inert conditions. We report a practical one-pot method that enables high-coverage NHC functionalization of gold nanoparticles using only imidazolium salts and a mild base. Systematic control experiments and spectroscopic analysis suggest a counterion-dependent mild-base activation pathway in which persistent HCO_3_^−^/CO_2_-derived intermediates are not observed under the applied reaction conditions. Instead, the data are most consistent with the formation of an intermediate that, in proximity to the nanoparticle interface, decomposes to form an Au–C bond. Optimized reaction conditions enable quantitative ligand exchange while suppressing particle ripening. The method is tolerant to air and moisture and is applicable to a broad range of NHC precursors, including benzimidazolium, imidazolinium, and mesoionic carbenes, as well as to different nanoparticle systems based on gold, silver, and copper. These results provide a general and mild strategy for preparing robust NHC-functionalized nanomaterials.

## Abstract 2

In this work, gold nanoparticles functionalized with N-heterocyclic carbenes are formed by a mild, one-pot activation with multiple NHC precursors. Mechanistic work rules out the formation of previously observed HCO_3_^−^ or CO_2_ adducts.

## Graphical abstract

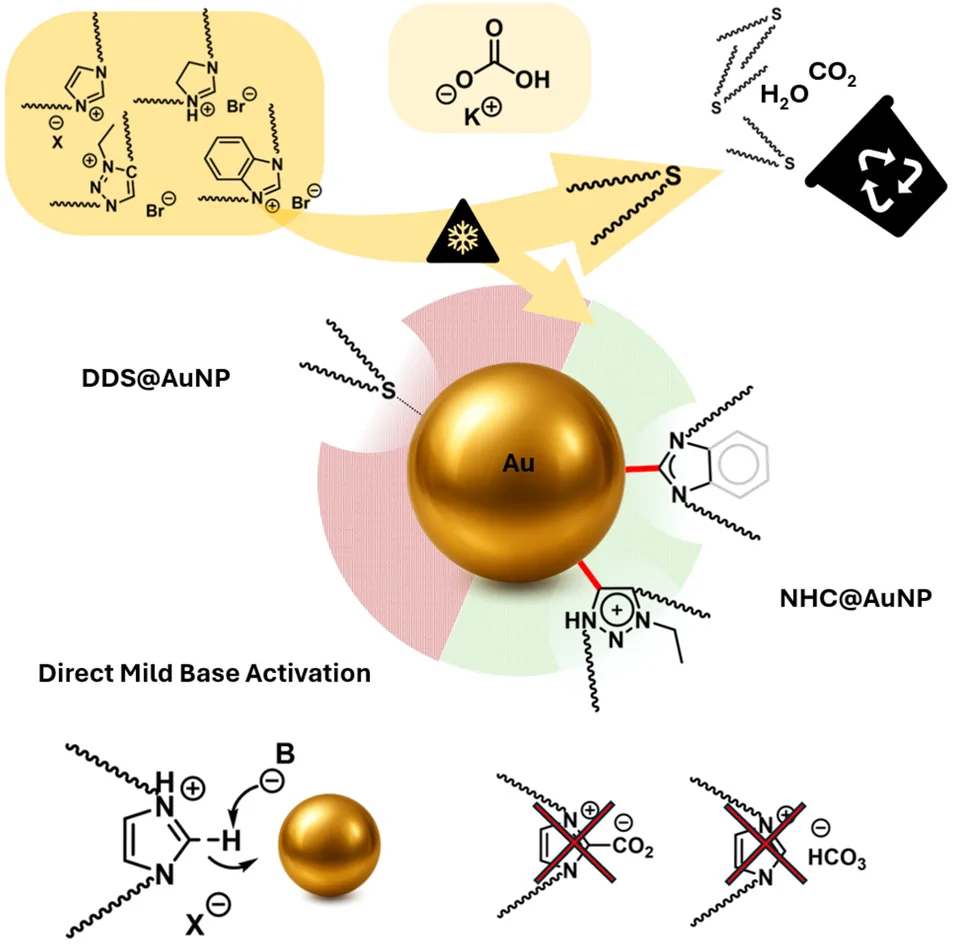

## Introduction

N-heterocyclic carbenes (NHCs) have emerged as powerful ligands for stabilizing gold nanoparticles (AuNPs) and various other surfaces.^1^ NHCs benefit from strong σ-donating properties and facile steric and electronic tunability, rendering them compelling alternatives to commonly used thiol-capping ligands.^2^ Additionally, the modular nature of NHC ligands enables the straightforward incorporation of a broad range of functional groups for diverse applications.^3^ In direct comparison, NHC-stabilized AuNPs (NHC@AuNPs) exhibit pronounced long-term stability with minimal surface leaching, even in biologically relevant media and under demanding heterogeneous catalytic conditions.^4^

Despite these advantages, the preparation of NHC@AuNPs can be challenging.^5^ NHC@AuNPs are mostly synthesized using two general routes, both of which are 2-step approaches: (1) the bottom-up (BU) approach involves the synthesis of a NHC metal complex, followed by reduction using strong reducing agents to form NHC@AuNPs;^6^ (2) the top-down (TD) approach is carried out through the generation of free^4*b*,6*a*^ or masked NHCs^7^ and subsequent ligand exchange with ligand-capped AuNPs. While BU approaches rely on reducing preformed NHC complexes, TD methods offer more flexibility by generating NHCs as surface-reactive species that can replace weaker ligands on metal surfaces. Unfortunately, the conventional TD synthesis of NHC@AuNPs is labor-intensive and usually requires multiple steps and equipment that allows for strictly inert conditions. Achieving a single-step and reliable TD installation of NHCs on the surface of AuNPs remains a significant challenge.^5*a*,8^

**Fig. 1:**
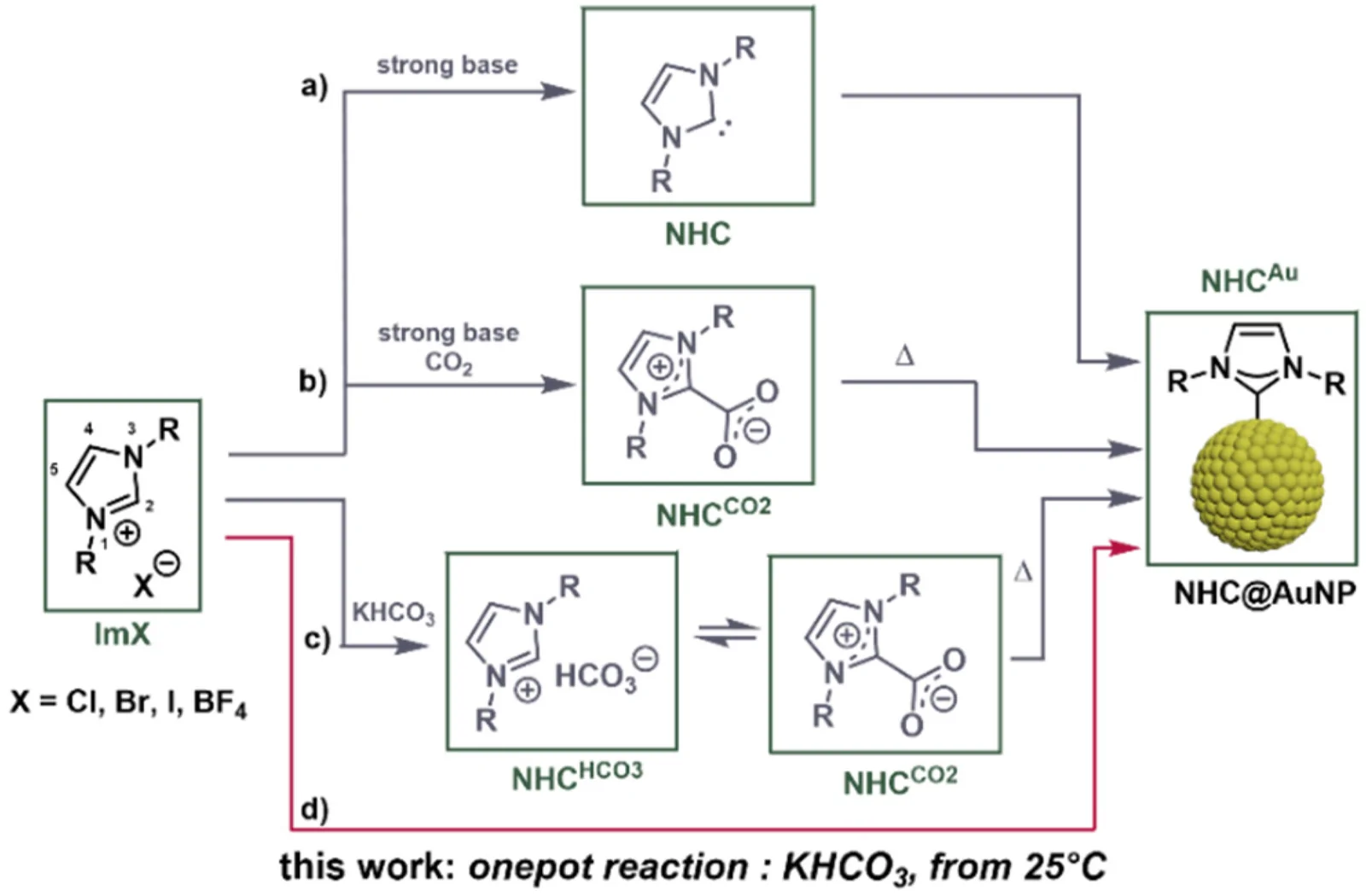
Comparison of the respective TD approaches toward NHC@AuNPs. While the most common route (a) relies on the free carbene, milder synthetic routes (b) and (c) use an NHC^CO_2_^ adduct. Our one-pot approach (d) results in quantitative ligand exchange from DDS@AuNPs to NHC@AuNPs.

Multiple groups have demonstrated successful ligand exchange using free NHCs generated with strong bases (Fig. 1a) and weaker ligand-stabilized AuNPs.^4*b*,6*a*,9^ However, such procedures require strongly basic conditions; therefore, more structurally elaborate NHCs with labile wingtip (N1/3) or backbone groups (C4/5) usually do not tolerate them and instead require milder NHC activation methods.^7*b*^

Similar to strategies used for the formation of NHC-metal complexes, the utilization of masked NHCs and the activation of NHC precursors with mild bases have been increasingly applied in surface and nanoparticle chemistry.^5*a*,7*b*,9*a*^ In the context of NHC surface modifications, NHC-CO_2_ adducts (NHC^CO_2_^) have been used to functionalize planar metal surfaces by heat-induced NHC activation and CO_2_ liberation.^9*a*^ Despite the comparably mild NHC activation, NHC^CO_2_^ adducts still require a strong base in order to generate the requisite carbene (C^2^)–CO_2_ bond and are therefore not compatible with more delicate NHC precursors (Fig. 1b).^10^ As an alternative to NHC^CO_2_^ adducts, Crudden *et al.* introduced NHC^HCO_3_^ as NHC precursors obtained by simple salt metathesis.^11^ Since in protic solvents NHC^HCO_3_^ and NHC^CO_2_^ are in equilibrium (Fig. 1c), the resulting mixture of NHC^CO_2_^/NHC^HCO_3_^ allows two possible pathways of NHC activation: the generation of NHCs by the liberation of CO_2_ when heated, and the NHC mild-base activation by the HCO_3_^−^ counterion.^12^ Using this concept, stable monolayers of NHCs on planar metal surfaces can be readily formed and have been used to successfully decorate AuNPs. Although salt metathesis using ion-exchange resins can be performed at ambient conditions, it still requires a multistep procedure and careful resin preconditioning.^11,13^ He and coworkers pioneered the use of NHC^CO_2_^/NHC^HCO_3_^ mixtures as NHC precursors on AuNPs, claiming that the surface reactive species is solely the NHC^CO_2_^ adduct.^14^

Additionally, Camden and co-workers reported an air-stable route to AuNPs *via* the combination of a molecular gold complex with citrate-stabilized AuNP.^15^ While this strategy is conceptually elegant, the synthesis of the gold complex is labor-intensive and necessitates additional purification steps. Furthermore, such complexes are typically costly when obtained commercially.

All described installations of NHC capping ligands on AuNPs are technically demanding and involve multistep syntheses (Fig. 1a–c). In this work, we aimed to establish a straightforward, one-step synthesis that avoids the need for specialized chemicals or complex equipment, as has been shown recently for bulk gold surfaces.^16^ The reaction conditions were optimized to establish a one-pot method for reproducible NHC@AuNPs (Fig. 1d), which are highly accessible and require only a vessel and inert gas. In addition, mechanistic investigations were conducted to gain insight into the binding and formation processes, thereby supporting the development of a practical and reproducible one-pot synthesis of NHC@AuNPs.

## Results and discussion

Despite the versatility of NHC ligands, their development and use have been constrained by the need for inert handling conditions, which poses a major barrier to their adoption compared with established thiol-based chemistry.

**Fig. 2:**
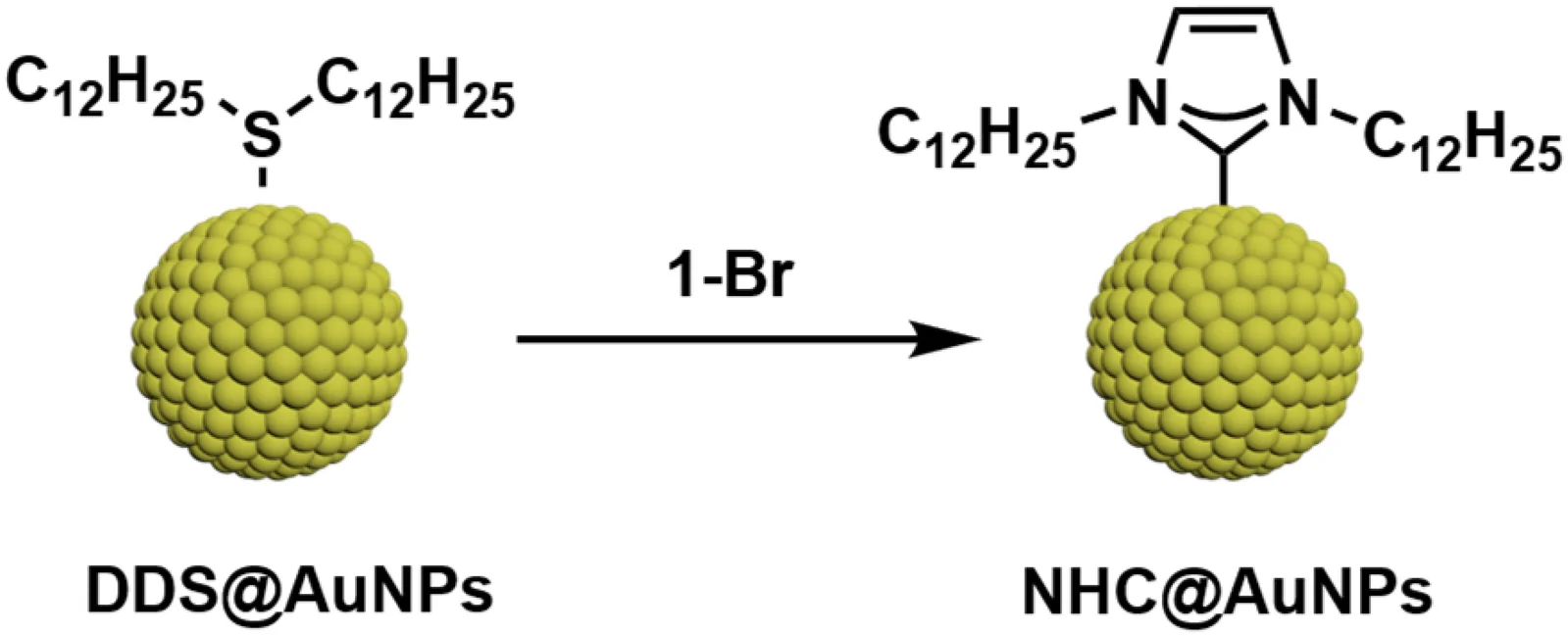
Reaction scheme for the one-pot ligand-exchange reaction. Reaction conditions were varied and are specified in Table 1.

We recently reported the direct ligand exchange between a PEG-functionalized imidazolium bromide ligand and oleyl amine-stabilized AuNPs in the presence of KHCO_3_.^7*b*^ Following this report, we aimed to develop a robust and versatile one-pot procedure for the TD synthesis of NHC@AuNPs. Optimization of the reaction includes screening different solvents, reaction temperatures, and the amount of the employed NHC ligand. All screening approaches were performed under inert conditions unless stated otherwise. The tested system comprises the common surface modifier for AuNPs, 1,3-didodecyl imidazolium bromide (1-Br), and DDS@AuNPs (Fig. 2).

DDS@AuNPs provide a reliable platform for monitoring ligand exchange reactions by analyzing high-resolution X-ray photoelectron spectroscopy (HR-XPS) spectra in the S 2p (175.4–157.4 eV) and N 1s (410.4–392.4 eV) regions.^6*a*^ Additionally, ligand exchange from sulfide-capped AuNPs is more challenging since they generally bind more strongly to the AuNP surface than phosphine or amine ligands, due to the hard–soft–acid–base (HSAB) theory. A successful exchange on such a demanding platform underscores the robustness of the applied reaction.^17^ Further, the formation of the Au–NHC bond can be verified in Raman spectroscopy by the red shift of the surface-bound N-heterocyclic ring stretch at ∼1400 cm^−1^ compared to the ImX precursor.^18^

DDS@AuNPs were synthesized according to an adapted literature-known procedure, resulting in narrowly dispersed particles with a size of 9 ± 3 nm and an organic content of 55 wt%, as determined by thermogravimetric analysis (TGA), both consistent with the literature (Fig. S1).^4*b*^ XPS analysis of the DDS@AuNPs showed a contribution at 163.3 eV, characteristic of surface-bound sulfides, and no signals in the N 1s region (Fig. S1 and S2).

The optimization of the one-pot reaction began by adapting conditions commonly reported for multistep procedures. Iterations for reaction optimization are listed in Table 1. 1-Br (13-fold excess based on DDS ligand estimated by TGA) and KHCO_3_ (14 eq.) were added to a dispersion of DDS@AuNPs in THF and heated to 40 °C for 72 h (Table 1, entry 1). The resulting dispersion was purified by passing through a syringe filter, followed by precipitation with acetone and redispersion in *n*-hexane to remove any unbound species and inorganic side products. XPS analysis of the sample showed successful NHC-binding to the AuNP surfaces, although residual sulfide moieties present in the S 2p spectrum indicate incomplete ligand exchange (Fig. S4).

**Table 1:** Screened reaction conditions for the optimization of the one-pot ligand exchange

Entry	Solvent	Base	*T* [°C]	*t* [h]	Eq.	Ligand exchange^a^
1	THF	KHCO_3_	40	72	13	Partial
2	Toluene	KHCO_3_	40	72	13	Partial
3	MeOH	KHCO_3_	40	72	13	Full
4	Dioxane	KHCO_3_	40	72	13	Full
5	Dioxane	KHCO_3_	40	2	13	Partial
6	Dioxane	KHCO_3_	40	24	13	Full
7	Dioxane	KHCO_3_	25	72	13	Full
8	Dioxane	KHCO_3_	5	24	13	Partial
9*	**Dioxane**	**KHCO**_**3**_	**25**	**24**	**13**	**Full**
10	Dioxane	KHCO_3_	25	24	8	Partial
11	Dioxane	KHCO_3_	25	24	4	Partial
12	Dioxane	Cs_2_CO_3_	25	24	13	Full
13	Dioxane	Pyridine	25	24	13	n.d.

a The ligand exchange is given as full (>90%), partial (<80%), and not observable (n.d.). In entry 9* optimized conditions can be found.

Based on these initial results, the impact of solvent polarity on exchange efficiency was studied. Ligand exchange experiments in two aprotic solvents of different polarity indices (*P*_I_), toluene (*P*_I_ = 2.4) and 1,4-dioxane (*P*_I_ = 4.8), as well as one protic solvent, MeOH (*P*_I_ = 5.1), were conducted. Based on S 2p XPS spectra (Fig. S4), toluene as a solvent led to incomplete ligand exchange (Table 1, entry 2). In contrast, the use of MeOH leads to nearly complete conversion, with only trace amounts of sulfide remaining on the particles (Table 1, entry 3 and Fig. S4), whilst 1,4-dioxane results in a complete ligand exchange, leaving no detectable sulfide ligands in the XPS (Table 1, entry 4 and Fig. S4). It should be noted that in these relatively polar solvents, particles slowly aggregate over the course of the reaction. Still, they were redispersible in *n*-hexane, as evidenced by the appearance of the dark red color and the characteristic plasmon band in the UV-Vis spectrum upon work-up (data not shown).

Following the successful exchange using 1,4-dioxane as the solvent, the reaction was further optimized by decreasing the reaction time from 72 h, with NPs isolated and characterized after 2 and 24 h (Fig. S5). Ligand exchange was incomplete after 2 h (Table 1, entry 5), as seen by the presence of the S 2p peak in XPS, but complete after 24 h (Table 1, entry 6) (Fig. S5). Interestingly, reaction time significantly influenced the particles’ morphology. After 72 h at 40 °C, significant ripening (TEM, 18 ± 4 nm, Fig. S4) resulted, while a reaction time of 2 and 24 h resulted in AuNPs with a smaller diameter compared to DDS@AuNPs (TEM, 6 ± 2 nm and 5 ± 1 nm, respectively, Fig. S5). The observed particle etching followed by ripening is likely due to the initial formation of NHC-Au adatoms, as reported previously.^19^ These adatoms can detach from the nanoparticle surface and enter the solution as free complexes.^19^ At longer reaction times, the subsequent size increase might be explained by redeposition of these complexes onto existing particles, thereby accelerating Ostwald ripening at elevated temperatures.^20^

Particle ripening is a major challenge in TD-based synthesis of NHC@AuNPs, as both reaction time and temperature critically affect the AuNP morphology. Therefore, lower reaction temperatures were explored for the NHC exchange, even though closely related systems reported in the literature typically require mild heating to release free NHC from NHC-CO_2_ precursors.^10^ Surprisingly, after 72 h, successful ligand exchange was also observed at 25 °C (Table 1, entry 7 and Fig. S7), and even partial ligand exchange was observed at 5 °C (Table 1, entry 8 and Fig. S7). TEM micrographs showed no significant ripening or etching at reduced temperatures (TEM (72 h, 25 °C), 8 ± 3 nm; TEM (72 h, 5 °C), 7 ± 2 nm) compared with the initial DDS@AuNPs (Fig. S7). Notably, full conversion was also observed when the reaction time was shortened to 24 h at 25 °C (Table 1, entry 9* and Fig. S6).

Although the optimized solvent system, reaction time, and temperature were established, the ligand exchange process remains influenced by the competitive adsorption of sulfide and NHC capping ligands on the nanoparticle surface. While the binding energy of NHCs to gold far exceeds that of DDS, favoring displacement, the existing DDS ligands can still desorb and re-adsorb transiently, maintaining an adsorption–desorption equilibrium.^6*a*^ Since DDS is released into solution upon exchange, complete conversion is only achieved when the imidazolium salt is present in large excess relative to the amount of DDS generated during the process. Consistent with this, lowering the indicial NHC precursor concentration below 13 equivalents relative to surface-bound DDS led to incomplete exchange (Table 1, entries 10 and 11, Fig. S8).

To probe the role of the base in the ligand exchange process, pyridine and Cs_2_CO_3_ were evaluated as alternatives to KHCO_3_ (Table 1, entries 12 and 13). Cs_2_CO_3_ afforded partial ligand exchange but induced pronounced particle etching, yielding AuNPs with a reduced average diameter of 3.2 ± 0.6 nm, consistent with UV-Vis changes (Fig. S9). KHCO_3_ was therefore retained due to its lower cost, accessibility, and higher exchange efficiency. Pyridine-treated samples showed an N 1s signal in XPS. However, this cannot be unambiguously assigned to ligand exchange due to possible contributions from adsorbed pyridine species. Raman spectroscopy revealed no characteristic NHC bands, indicating that pyridine does not promote effective exchange. Overall, these results demonstrate that carbonate bases are essential for successful ligand exchange under the investigated conditions.

**Fig. 3:**
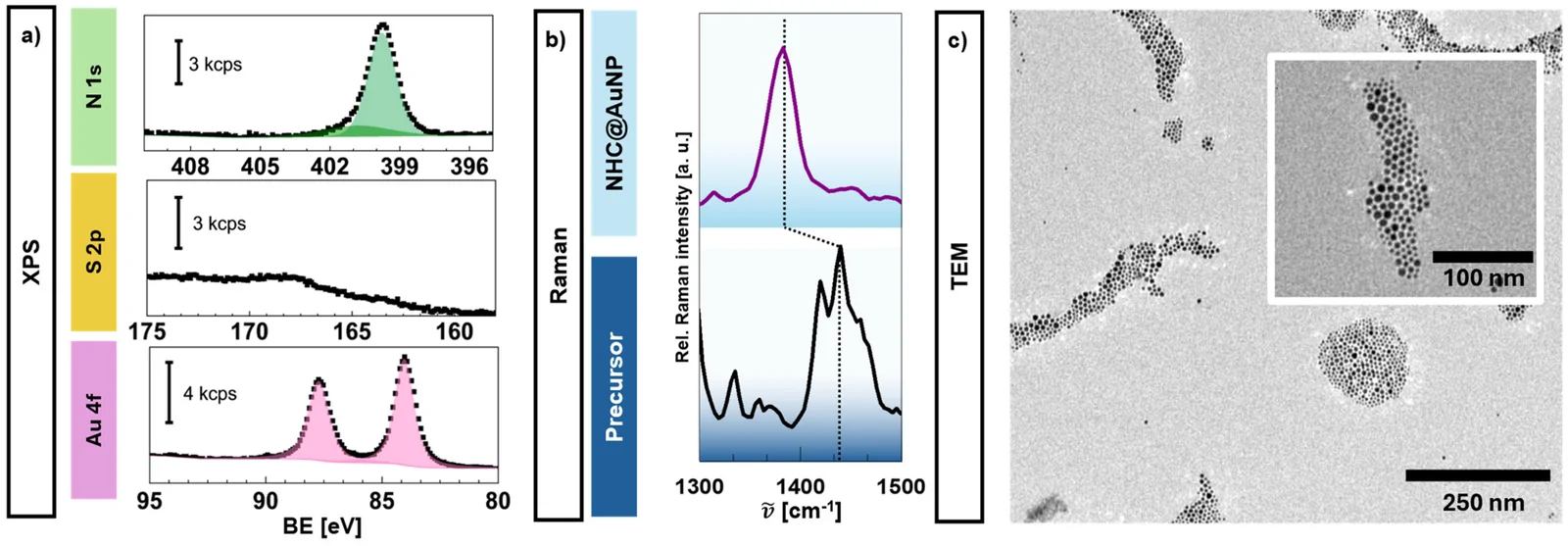
(a) High-resolution XPS analysis of the optimized NHC@AuNPs after ligand exchange. Shown here are N 1s, S 2p, and Au 4f spectra. (b) Analysis of the NHC@AuNP surface interaction using surface-enhanced Raman spectroscopy (SERS). (c). TEM micrographs of the NHC@AuNPs after successful NHC installation.

The final optimized conditions for the TD one-pot reaction were 1 : 13 : 14 DDS@AuNPs/1-Br/KHCO_3_ in 1,4-dioxane at 25 °C for 24 h under inert conditions. The resulting NHC@AuNPs were then further characterized by XPS, surface-enhanced Raman spectroscopy (SERS), UV-Vis, and TGA. XPS characterization of these particles revealed the absence of sulfide signals at ∼163 eV in the S 2p region and the presence of surface-bound NHCs at 400.7 eV in the N 1s spectrum (Fig. 3a). Characteristic signals for elemental gold were observed in Au 4f spectra for NHC@AuNPs (Fig. 3a). Interestingly, all tested reaction conditions and NHC precursors generated Au(0) [Au(0) 4f_5/2_ = 84.0 eV, Au(0) 4f_7/2_ = 87.7 eV, Au(i) 4f_5/2_ = 86.3 eV, Au(i) 4f_7/2_ = 90.0 eV]^21^ NP, except when the reaction time was only 2 h, as can be seen by two distinct gold species at 84.0 eV (Au(0)) and 84.9 eV (Au(i)) in the Au 4f spectra (Fig. S5). We attribute the presence of Au(i) at 2 h to the initial formation of a Au(i) NHC adatom, followed by its subsequent redeposition on the nanoparticle's surface over time (Fig. S1–S12).^15^ Particles did not show any signal in Br 3d XPS spectra, excluding ionic stabilization of KBr on the gold surface (Fig. S1). Furthermore, SERS of the obtained particles revealed the characteristic red-shift (*d*_Raman_ = 57 cm^−1^) of C–N stretch modes associated with the NHC structure upon binding to the AuNP surface (Fig. 3b and Fig. S15, S16). The characteristic C–N-Raman bands of NHC@AuNPs using 1-Br as surface ligand at 1382 cm^−1^ match reported values for other imidazole-based NHCs on AuNPs with different NHC wingtip modifications (lit. value for 1,3-diisopropylimidazolium capped AuNPs is 1401 cm^−1^) (Fig. 3b).^22^ TEM micrographs showed no significant ripening or etching compared to DDS@AuNPs (7 ± 2 nm, Fig. 3c).

Obtained particles also exhibited a localized surface plasmon resonance (LSPR) band at 512 nm, closely matching the absorbance maximum of DDS@AuNPs at 513 nm (Fig. S1). Using the LSPR as a marker of nanoparticle stability, the NHC@AuNPs remained stable for at least five weeks without any indication of decomposition (Fig. S10a). Their robustness was further evaluated by exposure to a 5 mM dodecanethiol solution for 60 h, during which no significant changes in the plasmon band were observed, indicating excellent resistance toward thiol-induced ligand displacement (Fig. S10b). Furthermore, under accelerated aging conditions in toluene at 100 °C, the NHC@AuNPs exhibited a lifetime approximately 12 times longer than that of the corresponding DDS@AuNPs (Fig. S10c and d). TGA measurements of the NHC@AuNPs indicated an organic content of about 36%, resulting in a high coverage of the particles with an estimated surface gold atom ratio of 1.1 (Fig. S1). This was in accordance with reports by Chechik and coworkers.^4*b*^ To further quantify the amount of NHC ligands bound to the nanoparticle surface, quantitative ^1^H-NMR (qNMR) analysis was performed. The NHC-functionalized AuNPs were digested with potassium iodide in dioxane at an elevated temperature until the complete disappearance of the plasmon resonance was observed, indicating full particle dissolution. The resulting dispersion was filtered to remove insoluble residues, and trimethoxybenzene was added as an internal standard for quantification. Owing to the complex digestion chemistry, several resonances originating from residual ligand-derived species were observed and included in the quantification. The qNMR analysis yielded an NHC ligand content of 32 wt% (Fig. S3), which is in excellent agreement with the value obtained by TGA, further supporting the conclusion of a complete ligand exchange and a densely packed NHC monolayer.

Furthermore, the robustness of the optimized reaction was demonstrated under different environmental conditions, namely, inert conditions (Schlenk), an air/argon mixture, and an air/argon mixture with the addition of water (Fig. S11). The reaction proceeded successfully under Schlenk conditions (Table 2, entry 1) and in the presence of air (Table 2, entry 2). In the presence of air, nearly quantitative ligand exchange was achieved, as observed by XPS, with only a minor fraction of residual unbound imidazolium salt (∼402 eV) and a strongly reduced contribution of thiol moieties (Fig. S11). In contrast, ligand exchange proceeding *via* a free carbene pathway typically requires a strictly inert atmosphere, highlighting the advantage of the one-pot approach. Notably, no further significant ripening was observed upon introduction of air, as observed by TEM (Fig. S11). Surprisingly, the addition of water resulted in nearly quantitative ligand exchange, as evidenced by the appearance of a signal at ∼400.5 eV in the N 1s XPS spectrum (Fig. S11 and Table 2, entry 3). This observation further suggests that the reaction proceeds predominantly through a pathway localized at the particle surface rather than *via* transient NHCs in solution, which would be rapidly protonated in the presence of water. However, water addition induced irreversible aggregation of the NHC-functionalized nanoparticles, due to the increased solvent polarity.

**Table 2:** Scope of the developed reaction using the optimized conditions on multiple materials. Air and water spiking of the reaction demonstrates its robustness

Entry	Precursor	Conditions	Gold material	Ligand exchange^a^
1	1-Br	Schlenk	DDS@AuNPs	Full
2	1-Br	Air-spiked	DDS@AuNPs	Full
3	1-Br	Water-spiked	DDS@AuNPs	Full
4	1-Cl	Optimized	DDS@AuNPs	Partial
5	1-I	Optimized	DDS@AuNPs	n.d.
6	1-BF_4_	Optimized	DDS@AuNPs	n.d.
7	2-Br	Optimized	DDS@AuNPs	Full
8	3-Br	Optimized	DDS@AuNPs	Partial
9	4-Br	Optimized	DDS@AuNPs	Full
10	1-Biss	Optimized	DDS@AuNPs	Partial
11	1-Mes	Optimized	DDS@AuNPs	Partial
12	1-Br	Optimized	Bulk gold	Deposition
13	1-Br	Optimized	OYA@AuNPs	Detectable
14	1-Br	Optimized	DDS@AgNPs	Full
15	1-Br	Optimized	OYA@CuNPs	Detectable

a The ligand exchange is given as full (>90%), partial (<80%), and not observable (n.d.).

**Fig. 4:**
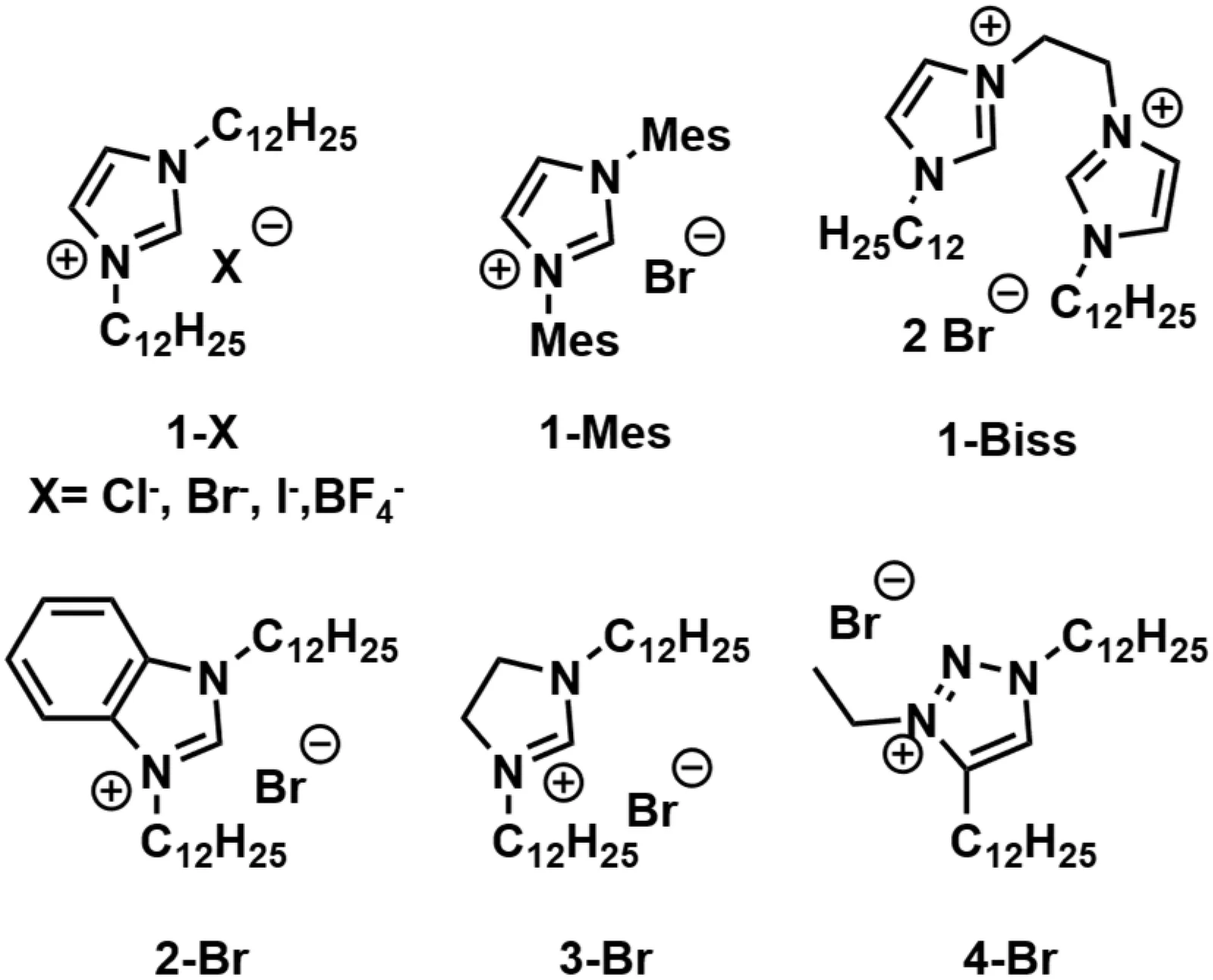
NHC and MIC precursors used in the optimized reaction conditions.

Upon optimization of reaction conditions, the structural elements and type of NHC precursor were modified to assess the versatility of the one-pot approach (Fig. 4). Initially, imidazolium counterions were considered due to their effect on the C^2^H proton acidity.^23^

To assess the influence of the counterion on the ligand exchange, the imidazolium salts 1-Cl, 1-Br, 1-I, and a non-coordinating 1-BF_4_ were investigated. While 1-Cl (Table 2, entry 4) and 1-Br (Table 1, entry 9*) afforded comparable ligand exchange under optimized conditions, no successful exchange was observed for either 1-I (Table 2, entry 5) or 1-BF_4_ (Table 2, entry 6). Instead, the resulting AuNPs exhibited a pronounced decrease in sulfide contributions in the S 2p XPS spectra (Fig. S12), while no corresponding NHC-related signal was detected in the N 1s region. These observations indicate that the original thiolate ligands are removed from the AuNP surface without concomitant installation of NHC ligands, yielding insufficiently stabilized nanoparticles that rapidly aggregate.

To rationalize these counterion-dependent differences in ligand exchange efficiency, we compared the imidazolium precursors based on literature reports, as the counterion is known to strongly influence the behavior of the C^2^H proton. In parallel, control experiments were conducted to assess the structural integrity of the nanoparticles in the presence of the respective salts in the absence of ligand. Previous studies have shown that the C^2^H/D exchange rate, a descriptor for the C^2^H proton mobility, and a downfield shift in the ^1^H-NMR spectrum of C^2^H, a descriptor of proton acidity, are strongly counterion-dependent.^24^ While fast proton exchange and a high chemical shift are observed for 1-Cl (10.9 ppm) and 1-Br (10.6 ppm), the exchange process is slower for 1-I (10.3 ppm) and is almost negligible for 1-BF_4_ (10.2 ppm) (Fig. 6 and S21).^24^ These trends are consistent with our observed ligand exchange efficiencies. Nevertheless, the influence of the 1-I and 1-BF_4_ on the system is evident from the substantial loss of sulfide ligand and the strong tendency toward aggregation. The counterions of the imidazolium salts are highly soluble in dioxane and can readily interact with the thiolate-decorated AuNP surface. Such interactions may arise from (i) dynamic adsorption of the counterions onto the nanoparticle surface, thereby inhibiting NHC coordination,^25^ and (ii) modification of the electrostatic environment at the interface, which can destabilize the existing thiolate layer.^26^

Together, these effects promote thiolate desorption without effective surface passivation, ultimately leading to aggregation. To evaluate the influence of the counterion, DDS@AuNPs were stirred under an inert atmosphere in dioxane at 25 °C for 24 h in the presence of KCl, KBr, KI, or KBF_4_. Owing to their limited solubility in dioxane, these salts provide a more controlled probe of counterion interactions with the nanoparticles. Structural changes were monitored by UV-Vis spectroscopy (Fig. S13), exploiting the sensitivity of the AuNP plasmon band to particle size and surface chemistry. A comparison with DDS@AuNPs stirred under identical conditions, but without added potassium salt, confirms that the observed changes arise from the presence of the salt rather than from stirring. Samples treated with KCl showed only minor shifts in the plasmon band and nearly unchanged full width at half maximum (FWHM), indicating that particle size and morphology were preserved. KBr and KBF_4_ induced a slight redshift with FWHM broadening, whereas KI caused the largest shift and the broadest FWHM. Moreover, the overall particle concentration decreased in the KBr and KI-containing samples, indicating particle loss. Since all experiments were performed at identical initial concentrations, the absorbance is expected to remain constant in the absence of aggregation (Fig. S13).

These results align with established reports on halide-induced poisoning and etching of gold surfaces, which follow the trend I > Br > Cl^27^ as well as with the disruptive influence of BF_4_ ^26^ on surface-bound ligands. Iodide, as the softest halide (HSAB concept), interacts most strongly with the soft Au surface through favorable orbital overlap. This leads to the most effective blocking of possible anchoring sites, preventing ligand exchange. Bromide, in contrast, binds moderately while leaving sufficient surface sites accessible, resulting in the most efficient ligand exchange. Although weakly adsorbing, chloride provides less surface stabilization, reducing exchange efficiency. Thus, efficient ligand exchange requires counterions whose interactions with Au are sufficiently weak to avoid surface-site blocking while still allowing thiolate displacement, thereby providing the conditions for NHC installation.

**Fig. 5:**
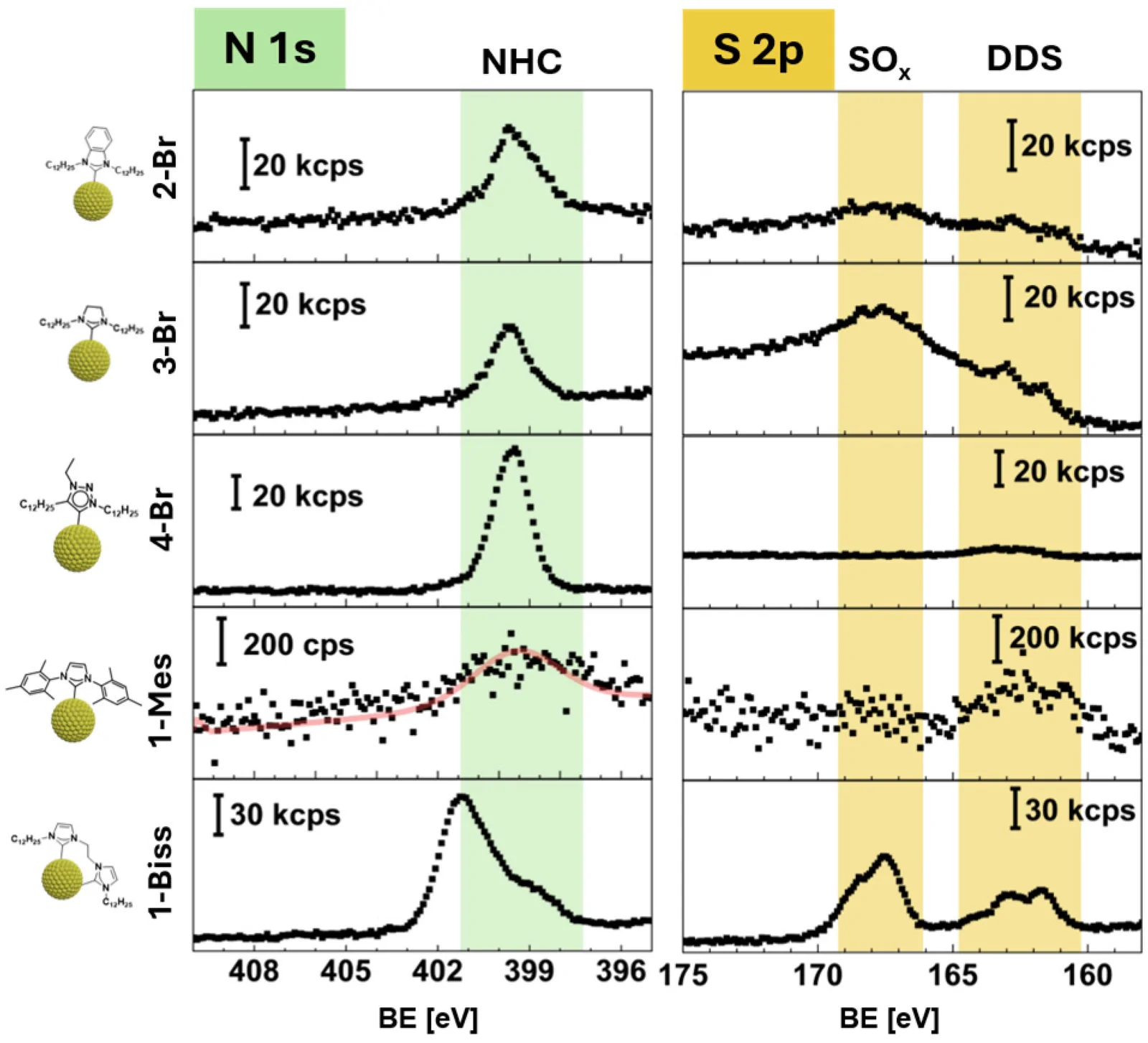
XPS analysis of various NHC/MIC@AuNPs after ligand exchange further verified the complete ligand exchange by the disappearance of the thiol signal in the S 2p scan at ∼162 eV, while clear NHC signals in the N 1s at ∼400.5 eV spectrum can be observed.

To further test the reaction's robustness, we investigated a series of NHC starting materials, including benzimidazolium (2-Br, Table 2, entry 7), imidazolinium (3-Br, Table 2, entry 8), and meso-ionic carbenes (MICs), such as 1,2,3-triazolium (4-Br, Table 2, entry 9) bromides, each with identical wingtip functionalization (Fig. 4 and Fig. S14). Applying the optimized conditions for ligand exchange with DDS@AuNPs for all ligands led to successful ligand exchange with a characteristic surface-bound carbene signal at ∼400.5 eV in the respective N 1s XPS spectra with varying exchange efficiency (Fig. 5). While 2-Br and 4-Br exhibited complete exchange, 3-Br showed small amounts of residual dodecyl sulfide. Additionally, SERS measurements revealed characteristic red shifts of the C^2^–N bands, further confirming successful carbene surface-bonding (Fig. S15 and S16). TEM micrographs of AuNPs capped with the NHCs derived from 3-Br (5 ± 1 nm) and 4-Br (5 ± 2 nm) showed etched particles (Fig. S14) when compared to DDS@AuNPs. However, NHC@AuNPs using 2-Br underwent no size change, resulting in particles with a size of 9 ± 4 nm (Fig. S14). Since NHCs are strong σ-donors, they can destabilize the local Au lattice, pull adatoms out of the surface, and in some cases even remove individual Au atoms into solution.^19^ The efficiency of this atom-lifting process aligns with the σ-donor strength of the carbene. The benzimidazole-based carbene 2-Br exhibits strong *p*-delocalization of the carbene lone pair, which reduces its σ-donor strength; this results in the weakest tendency to extract the Au atom and thus produces the smallest overall etching effect.^28^ Furthermore, imidazolium bromide ligands with different wingtip modifications were tested, including 1-Biss (Table 2, entry 10) as a multidentate ligand and 1-Mes (Table 2, entry 11) with more steric hindrance close to the C^2^ position. Tolerance toward different wingtip modifications was confirmed through the observation of characteristic surface-bound NHC peaks at ∼400 eV (Fig. S17) in the N 1s XPS spectra. However, both samples showed incomplete conversion. In the case of 1-Biss, the bidentate architecture is strained due to the short linker, giving the ligand only limited conformational flexibility. This constraint can result in the bidentate unit binding only through one carbene site, thereby blocking neighbouring surface positions and preventing full ligand exchange. The incomplete substitution is reflected in the free imidazolium contribution (401.7 eV) observed in the N 1s spectra and the residual signal in the S 2p spectra. For 1-Mes precursors, the C^2^H proton is sterically shielded, which reduces adsorption on the particle's surface.^19^ Under weakly basic conditions, this leads to a lower extent of conversion and thus incomplete formation of the surface-bound NHC.

The model compound 1-Br was also applied to bulk gold surfaces and under the optimized conditions (10 mM, 1-Br in dioxane, 24 h, 25 °C, Table 2, entry 12). Bulk gold surfaces were achieved by physical vapor deposition (PVD) on a glass slide. The appearance of a signal in N 1s XPS analysis at 400.5 eV confirmed successful NHC binding to the gold surface (Fig. S18). HR-XPS spectra of the Br 4d region indicated no bromide contamination on the gold surface, demonstrating that the method is applicable not only to nanoparticles but also to bulk surfaces, as recently shown.^16^

In a final step, the optimized ligand exchange conditions were applied to nanoparticle systems with different parent ligands and metal cores, namely oleylamine-stabilized AuNP (OYA@AuNPs), didodecyl sulfide-stabilized silver nanoparticles (DDS@AgNPs), and oleylamine-stabilized copper nanoparticles (OYA@CuNPs) (Table 2, entries 13–15). OYA@AuNPs were selected to demonstrate the applicability of the protocol to alternative AuNP substrates, DDS@AgNPs to evaluate its transferability to AgNPs, and OYA@CuNPs to extend the methodology to CuNPs. Notably, DDS@AgNPs gradually convert into Ag_2_S nanoparticles upon storage, making the use of freshly prepared AgNPs essential.^29^ As the analogous conversion of sulfide-stabilized CuNPs to Cu_2_S proceeds considerably faster, only oleylamine-stabilized CuNPs were investigated.^30^ Successful ligand exchange was confirmed for all systems by the appearance of the characteristic NHC-derived SERS bands (Fig. S19). However, monitoring the reaction by XPS depended on the nature of the parent ligand. In the case of DDS@AgNPs, the disappearance of the S 2p signal originating from the sulfide ligand enabled direct observation of complete ligand exchange (Fig. S18). Furthermore, TEM analysis revealed no evidence of nanoparticle ripening during the exchange process, with the particle size remaining essentially unchanged from 4.8 ± 1.4 nm for DDS@AgNPs to 4.6 ± 1.5 nm for the corresponding NHC@AgNPs (Fig. S18).

For the oleylamine-stabilized Au- and CuNPs, direct monitoring of the ligand exchange by XPS was not possible due to the absence of a sulfide moiety giving rise to an S 2p signal. Consequently, confirmation of successful ligand exchange relied primarily on the SERS measurements, which clearly showed the characteristic vibrational features of the coordinated NHC ligands. In addition, the XPS spectra exhibited characteristic binding-energy shifts and spectral changes observed for the other NHC-functionalized AuNPs described in this work and for NHC-functionalized CuNPs reported in the literature, providing further evidence of successful surface functionalization.^31^ TEM analysis further demonstrated that CuNPs underwent ripening during the ligand exchange process, with the average particle sizes increasing (Cu: 3.4 ± 1.1 nm to 6 ± 2 nm, Fig. S18).

Throughout the optimization process of this one-pot method, insights into the imidazolium activation mode have been identified, including the influence of the reaction conditions, solvent system, and N-heterocycle counterions. Notably, the partial installation of NHC ligands at 5 °C suggests a different activation path than the previously reported route *via* NHC^CO_2_^ adducts as the active species.^32^ Given the thermal stability of NHC^CO_2_^ adducts below 12 °C, a mild base-mediated ligand transfer appears to be much more probable.^14^ To determine whether the reaction proceeds in the absence of a base, a control reaction was performed under identical conditions in the absence of a base. XPS analysis of this control experiment showed no N 1s signal, indicating that the reaction does not proceed without the presence of KHCO_3_ (Fig. S11).

Previous reports suggested thermal activation of the masked NHC^CO_2_^ adduct as the predominant source of surface-bound NHC.^14^ MeOH, as a protic solvent, has been reported to shift the equilibrium towards NHC^HCO_3_^ ^7*b*^ whereas 1,4-dioxane suppresses the equilibrium. Additionally, pure NHC^CO_2_^ adducts are stable at 12 °C and, therefore, should not release surface-reactive NHC below this threshold. Since a partial ligand exchange was still observed at temperatures as low as 5 °C, a ligand exchange using an NHC^CO_2_^ adduct-dependent pathway may only partially account for the mechanism.

**Fig. 6:**
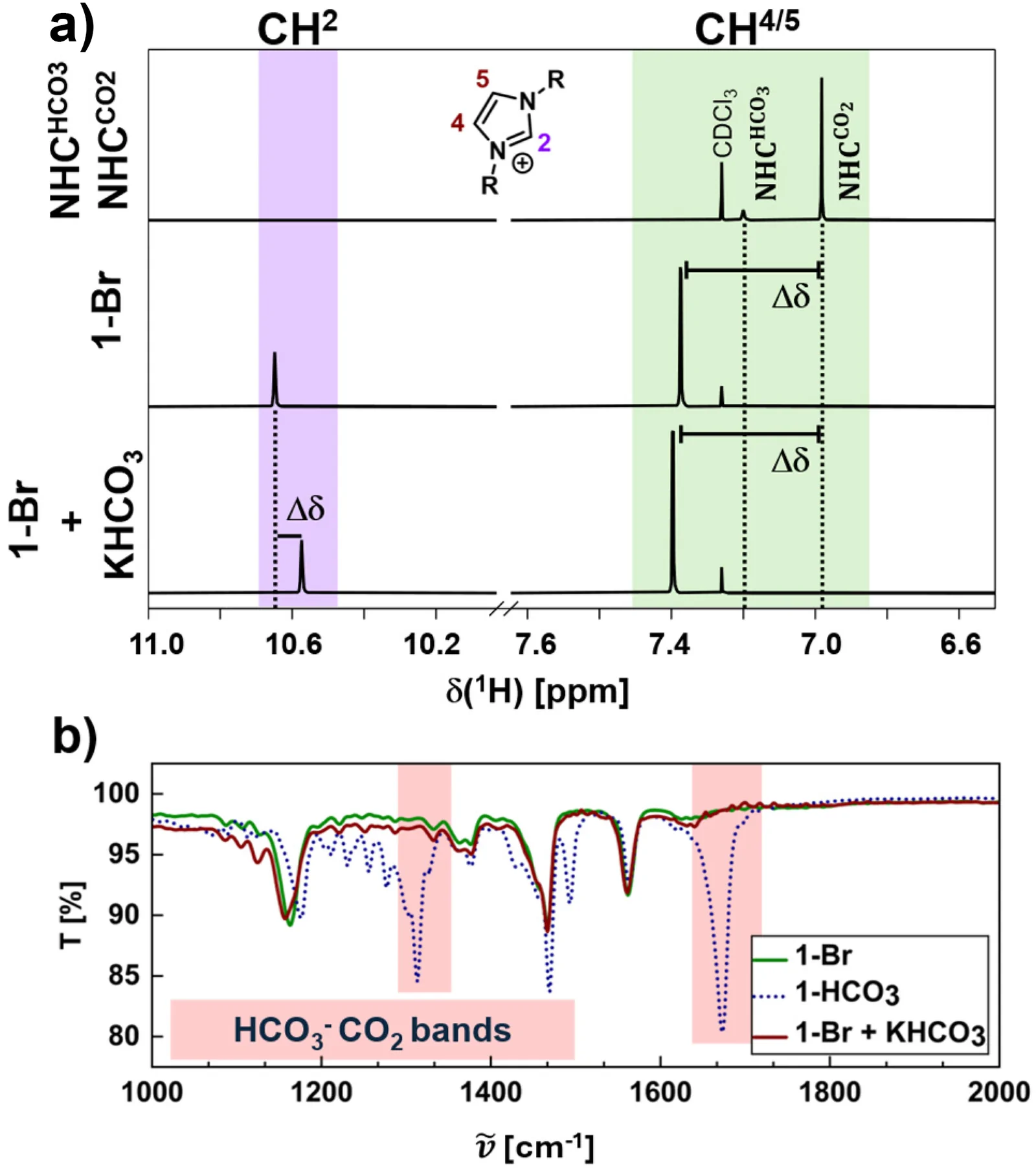
(a) ^1^H NMR studies of the electronic properties of treated 1-Br, untreated 1-Br, and NHC^HCO3^/NHC^CO2^. Comparison of treated 1-Br with untreated 1-Br showed significant downfield shifts in the C2 signal. Comparing C4/5 signals from 1-Br-treated samples to those from NHC^HCO3^/NHC^CO2^ shows no signs of salt metathesis. All spectra were recorded in 0.7 mL of absolute CDCl_3_ using 20 mg of the substance and 4.4 mg of KHCO_3_ to exclude concentration effects. After the reaction time, the suspension was filtered, and the solvent was removed under reduced pressure. (b) Additional comparison of IR-spectra of 1-Br, NHC^HCO3^/NHC^CO2^ mixture, and treated 1-Br further verified the absence of salt metathesis in dioxane at room temperature.

In an attempt to get a better understanding of the reaction pathway, 1-Br (1 eq.) was stirred with KHCO_3_ (1.1 eq.) in 1,4-dioxane for 24 h at 25 °C, simulating the reaction conditions applied to the AuNPs. The mixture was then filtered to remove the influence of unreacted KHCO_3_, and the solvent evaporated. To avoid unwanted concentration effects, all spectra were recorded using the same amount of 1-Br and KHCO_3_. The obtained white solid, suspected to be the active intermediate, was analyzed by ^1^H-NMR in CDCl_3_ (abs.), high-resolution mass spectroscopy (HRMS), and Fourier transform infrared (FTIR) spectroscopy. FTIR and NMR spectra of treated 1-Br were compared to a pre-made mixture of NHC^CO_2_^/NHC^HCO_3_^ by the reaction of the free NHC with CO_2_ (Fig. 6). Although CO_2_ usually affords only the NHC^CO_2_^ adduct, minor amounts of NHC^HCO_3_^ were present, likely due to trace moisture in the CO_2_ gas. To maintain the possible NHC^CO_2_^/NHC^HCO_3_^ equilibrium, any moisture was carefully excluded from all samples and measurements.

Comparing the C^4/5^ region between the NHC^CO_2_^ adduct and the 1-Br treated with KHCO_3_ in 1,4-dioxane confirmed the absence of NHC^CO_2_^ species. Further comparison of the C^2^H proton also reveals the absence of NHC^HCO_3_^ species. The comparison with untreated 1-Br showed a downfield shift (*δ*Δ = 0.08 ppm) of the C^2^H signal, indicating a change in the proton acidity.^24^ Interestingly, this shift becomes more pronounced over the course of the reaction and stabilizes after 12 hours, highlighting the importance of sufficient reaction time (Fig. S20).

Treatment of 1-Cl with KHCO_3_ in dioxane for 24 h caused a comparable downfield shift of the C^2^H resonance (*δ*Δ = 0.11 ppm), although the overall C^2^*H* intensity was reduced due to accelerated H/D exchange. Interestingly, Cl^−^ induced C^2^H lability was sufficient to form a small amount of NHC^CO_2_^, as verified by a ^1^H-NMR signal at 7.01 ppm (Fig. S21).

Applying the same reaction conditions to 1-I and 1-BF_4_ did not lead to comparable downfield (respective *δ*Δ = 0.01, 0.03 ppm) shift of the C^2^H proton or NHC^CO_2_^ buildup, indicating insufficient interaction between ImX and the base (Fig. S21). To further prove that 1-Br relies on a mechanism without NHC^HCO_3_^ or NHC^CO_2_^, HRMS and FTIR of treated 1-Br were performed. This revealed no bicarbonate mass fragments, further supporting the absence of ion metathesis under the applied conditions (Fig. S22). Comparison of the FTIR spectrum of the mixture of NHC^CO_2_^/NHC^HCO_3_^ with the treated 1-Br also showed no signs of salt metathesis, but rather an identical fingerprint of untreated 1-Br (Fig. 6b).

**Fig. 7:**
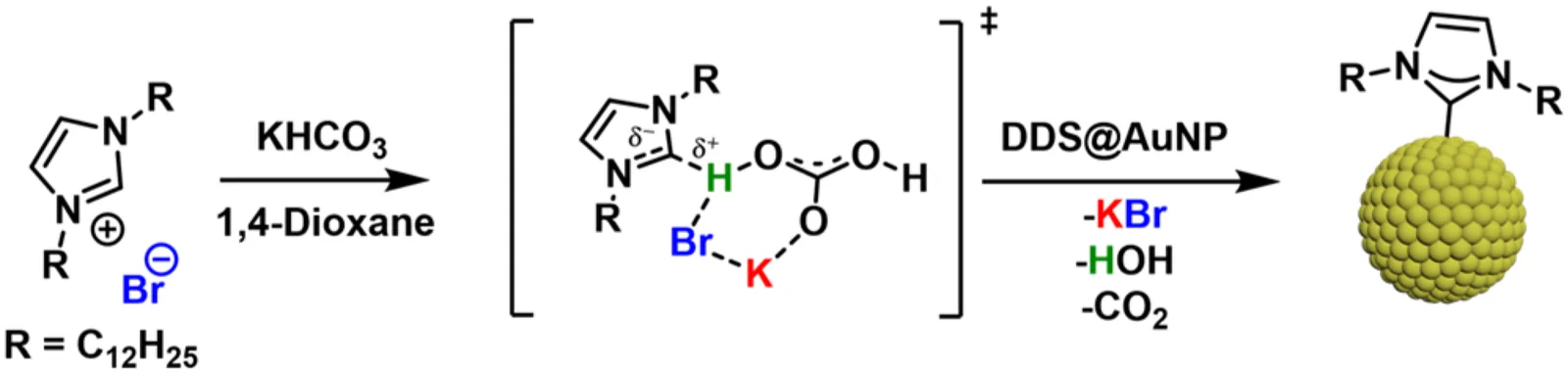
Proposed concerted mechanism for NHC ligand installation on AuNP, in which mild base-mediated deprotonation of the imidazolium salt and subsequent rapid *in situ* NHC coordination at the gold surface occurs. The configuration of the transient state is approximately derived from the experimental results.

While the present data do not allow the assignment of a single elementary reaction pathway, several mechanistic aspects can nevertheless be inferred from the experimental observations. Under the applied reaction conditions, no NHC^CO_2_^ or NHC^HCO_3_^ intermediates were detected by IR spectroscopy. Furthermore, partial ligand exchange was already observed at 5 °C (Fig. S6), making a pathway involving NHC^CO_2_^ adducts less likely, as these species generally require elevated temperatures to release the free carbene. The C^2^H chemical shift observed in the ^1^H NMR spectra (Fig. S21), together with the absence of ligand exchange in the absence of base (Fig. S11), is consistent with KHCO_3_ acting as a weak base to facilitate deprotonation of the imidazolium precursor. Finally, the pronounced counterion dependence (Fig. S21) suggests that the hydrogen-bonding environment of the C^2^H proton plays an important role in the reaction. Taken together, these observations are consistent with an activated imidazolium species occurring in close proximity to the Au surface, where it is rapidly captured by the nanoparticle. While alternative mechanistic pathways cannot be excluded on the basis of the present data, the proposed surface-assisted deprotonation and capture pathway (Fig. 7) provides a plausible explanation for the observed experimental results.

## Conclusions

Until now, most protocols for installing NHCs on AuNPs have relied on two-step approaches that require specialized equipment and stringent inert conditions. We report the straightforward, robust one-pot installation of NHC using an imidazolium salt and a mild base. Through systematic screening of solvent, temperature, equivalents, and counterions, optimal conditions for the one-pot NHC installation were identified: 25 °C, 24 h, 13 equivalents of NHC precursor, and a Br^−^ counterion, affording complete ligand exchange. This procedure proved broadly applicable across different NHC systems, including different wingtip modifications and four distinct carbene classes. Successful ligand exchange of different nanoparticle classes and surface functionalization were confirmed by XPS and Raman spectroscopy, while particle stability and morphology were verified by UV-Vis spectroscopy and TEM.

Additionally, our spectroscopic analysis supports a mild base-mediated, counterion-dependent activation pathway in which persistent NHC^CO_2_^ intermediates are not observed under the applied reaction conditions. NMR data indicate that the process is initiated by a destabilization of the C^2^H bond through the counterion and potassium bicarbonate. Subsequent decomposition of this intermediate may, upon spatial proximity to the AuNP surface, form NHC@AuNPs. In contrast to conventional protocols, this method does not require an inert atmosphere or specialized equipment. Remarkably, the reaction tolerates air and even water, though the particles’ structural integrity is compromised in the latter case. The simplicity, robustness, and versatility of this approach position it as a significant advancement in the field, making it broadly applicable to researchers without specialized equipment and enabling faster progress.

## Author contributions

RR and CE contributed to conceptualization, investigation, methodology, formal analysis, data curation, writing, reviewing and editing, visualization. SRT contributed to writing, reviewing and editing and formal analysis. JCM contributed to conceptualization, funding acquisition, resources, writing – review and editing, formal analysis. MRR contributed to conceptualization, funding acquisition, writing, reviewing and editing, formal analysis, project administration, resources and supervision.

## Conflicts of interest

There are no conflicts to declare.

## Supplementary Material

NR-018-D6NR01951J-s001

## Data Availability

All experimental data supporting this study are provided in the supplementary information (SI). Supplementary information: complete synthetic procedures, characterization data, and optimized reaction conditions, comprehensive ligand scope and substrate screening, detailed particle characterization *via* XPS, TEM and UV-Vis spectroscopy, SERS analysis of model compound 1-Br and other NHC precursor materials, and mechanistic studies comprising time-resolved *in situ* NMR spectroscopy and high-resolution mass spectrometry of NHC precursors with various counter ions. See DOI: https://doi.org/10.1039/d6nr01951j.
