# Supplementary material for: One-pot installation of N-heterocyclic carbenes on gold nanoparticles through counterion-dependent mild-base activation
Source: Nanoscale. 2026 Sep 2;18(38):20863–72. doi: 10.1039/d6nr01951j (PMC13563439; doi:10.1039/d6nr01951j)
Supplement: Supplementary file 1 [file NR-018-D6NR01951J-s001.pdf]

Supplementary Information (SI) for Nanoscale.
This journal is © The Royal Society of Chemistry 2026

One-Pot Installation of N-Heterocyclic Carbenes on Gold Nanoparticles through Counterion-Dependent Mild-Base Activation

Robert Richstein,[a,c] Constantin Eisen,[a,b] Sophie R. Thomas,[a] Jia Min Chin,*[b,d] and Michael R. Reithofer*[a]

[a] R. Richstein, C. Eisen, S. R. Thomas, M. R. Reithofer
Institute of Inorganic Chemistry, Faculty of Chemistry
University of Vienna
Währinger Str. 42, 1090 Vienna, Austria
E-mail: michael.reithofer@univie.ac.at

[b] C. Eisen, J. Chin
Institute of Functional Materials and Catalysis, Faculty of Chemistry
University of Vienna
Währinger Str. 42, 1090 Vienna, Austria
E-mail: jiamin.chin@univie.

[c] R. Richstein
Vienna Doctoral School in Chemistry
University of Vienna
Währinger Str. 42, 1090 Vienna, Austria

[d] J. Chin
Wolfgang-Pauli-Institute
Oskar-Morgenstern-Platz 1
1090 Vienna, Austria

Materials and Methods

All experiments, if not stated otherwise, were performed using standard Schlenk technique or an argon (Ar) filled MBraun Unilab Pro glovebox using dry solvents and flame-dried glassware. Commercially available reagents were used without further purification. Dry solvents were obtained from Sigma Aldrich or Acros/Fisher and stored over activated molecular sieves (3 Å) inside a glovebox. Deuterated solvents ($CDCl_3$ and DMSO-d6) were obtained from Sigma Aldrich. All molecular products were fully characterized using multinuclear NMR spectroscopy and high-resolution mass spectrometry. Obtained nanomaterials were characterized using XPS, UV-Vis, TGA, TEM, and Confocal Raman spectroscopy.

Nuclear Magnetic Resonance spectra (NMR) were recorded on either a Bruker BioSpin AV III 600 (^{1}H-NMR: 600.25 Hz, ^{13}C-NMR: 150.93 Hz) or Bruker BioSpin AV neo 500 (1H-NMR: 500.32 Hz, ^{13}C-NMR: 125.81 Hz) provided by the NMR Center, Faculty of Chemistry, University of Vienna. The residual solvent peaks were used as a reference. The resulting spectra were processed using TopSpin v4.1.1 and analyzed with MestReNova v14.1.2.

Mass spectrometry (MS) was performed on a Brucker amaZon speed ETD (reaction control/routine measurements) or Bruker maXis UHR-TOF (high resolution) spectrometer at the Mass Spectrometry Centre, Faculty of Chemistry, University of Vienna

Confocal Raman spectra were acquired using a WITec alpha300 A confocal Raman microscope equipped with a 532, 633, 785 nm laser, and a high-throughput spectrometer coupled to a cooled CCD detector (DU401_Br, DD).
Nanoparticles were aggregated by the addition of 10 mL of methanol to a concentrated dispersion of the nanoparticles in a minimal amount of hexane. The suspension was allowed to sit for 1 h before the black aggregates were drop-cast onto freshly cleaned silicon wafers and allowed to dry under reduced pressure.
Measurements were performed using a Zeiss LD EC Epiplan-Neofluar Dic 50x / 0.55. The laser power at the sample was set to a maximum of 5 mW to avoid photodamage. Spectra were recorded with an integration time of 100 s, averaged over 20 accumulations.
The system was calibrated using the Raman band of a silicon wafer at 520.7 cm^{-1} before measurements. All spectra were processed using WITec Project software (version 7.0). Baseline correction and smoothing were performed using OriginPro 2020b.

Thermogravimetric analysis (TGA) was performed on a TGA/DSC 3+ STARe Thermal Analysis System Jupiter under a nitrogen flow of 10mL/min. 1-5 mg of nanoparticles were dispersed in toluene and drop-cast into a pre-weighted 70 µL crucible. The solvent was then evaporated at 100°C for 48 h. Completion of the drying process was monitored using a precision balance. All measurements were started after a constant weight was reached. In a typical measurement, the particles were heated to 110 °C for 2 h before starting the measurement from 110 to 800 °C at 10 °C/min.

X-ray photoelectron spectroscopy (XPS) was performed on a Nexsa Photoelectron Spectrometer (Thermo Fisher Scientific, UK) provided by the Core Facility "Interface Characterization", Faculty of Chemistry, University of Vienna. Samples were drop-cast from dispersions in toluene onto a freshly cleaned silicon wafer and dried for 24 h at elevated temperature. The silicon wafers were cleaned by sonication in methanol and acetone, followed by drying under an argon stream. Element specific high-resolution spectra for Carbon (C 1s 279-298 eV), Nitrogen (N 1s 392-410 eV), Sulfur (S 2p 157 - 175 eV), Bromine (Br 3d 58 - 82 eV), Chlorine (Cl 2p 190 – 210 eV), Iodine (I 3d 610 – 640 eV) and Gold (Au 4f 79 – 99 eV) were recorded with step size of 0.1 eV and a pass energy of 50 eV using 30 scans. All measurements were performed using Al-Kα X-rays with a spot size of 400 µm. Obtained spectra were evaluated using the Advantage software package v5.9929 provided by Thermo Fisher Scientific and Origin Pro v9.7.5.184

UV-Vis spectroscopy was conducted on an Agilent Technologies G1103A using quartz cuvettes. Nanoparticles dispersible in apolar solvents were measured in hexane, while water-soluble nanoparticles were measured in water. Before each measurement, the solvent (hexane or water) was measured as background.

Transmission electron microscopy (TEM) was measured at the Core Facility Cell Imaging and Ultrastructure Research, University of Vienna - member of the Vienna Life-Science Instruments (VLSI) using a ZEISS Libra 120 (120kV) TEM equipped with a CCD Camera Sharp Eye TRS. Prior to measurement, samples suspended in toluene were drop-cast onto formvar-coated carbon film on 200 mesh copper grids. The resulting pictures were processed, and the average particle diameter (d) and size distribution were analyzed with ImageSP Version 1.2.71.

Procedure P1 Synthesis of (Benz)-Imidazolium Salts

(Benz)-Imidazolium halides were synthesized according to an adapted procedure from Mebrouk et al.[1]

In short, benzimidazole or imidazole (15 mmol, 1 eq.) was dissolved in absolute ACN (20 mL), and potassium hydroxide (1.5 eq.) was added, and the mixture was stirred for 20 min at RT. After slow addition of alkylation agent (1.1 eq.), the reaction mixture was stirred for 16 h at RT. The final mixture was filtered, and the filtrate was concentrated under reduced pressure. The resulting monoalkylated intermediate was dissolved in ACN (20 mL), and the alkylation agent (2 eq.) was added. The resulting solution was heated to 95 °C for 24-48 h, cooled to RT, and concentrated under reduced pressure. The solid residue was dissolved in a small amount of DCM, and the addition of cold diethyl ether afforded the (benz)-imidazolium salts upon collection by centrifugation and drying in vacuo.

Imidazolium tetrafluroborates were synthesized according to an adapted procedure from Huynh et al.[2]

In short, imidazolium bromide (15 mmol, 1 eq.) was dissolved in acetone (20 mL), and potassium tetrafluoroborate (2 eq.) was added, and the mixture was stirred for 16 h at RT. All volatiles were removed under reduced pressure before the addition of DCM. The resulting suspension was filtered over celite, and drying in vacuo yielded the title compounds.

1,3-didodecyl imidazolium chloride - **1-Cl**

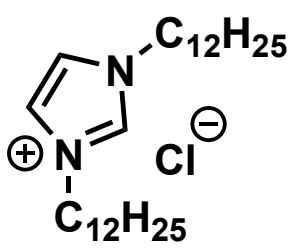

1-Cl
441.19 g/mol
$C_{27}H_{53}N_2Cl$

1,3-didodecyl imidazolium chloride (5.8 g, 13.1 mmol, 90%) was obtained by the reaction of imidazole (1 g, 14.7 mmol, 1 eq.), KOH (1.24 g, 22.1 mmol, 1.5 eq.), and 1-chlorododecane (3.8 mL, 16.1 mmol, 1.1 eq. and 6.9 mL, 29.4 mmol, 2 eq.) in ACN.

^{1}H-NMR (500 MHz, $CDCl_3$) δ = 10.87 (s, 1 H), 7.31 (d, J = 1.5 Hz, 2 H), 4.33 (t, J = 7.4 Hz, 4 H), 1.92-1.86 (m, 4 H), 1.30-1.22 (broad, 36 H), 0.85 (t, J = 7.0 Hz, 6 H) ppm. Spectroscopic data matches previously published data.[3]

m/z (ESI) Calcd. for $C_{27}H_{53}N_2Cl$ [M-nCl, $C_{27}H_{53}N_2^+$]: 405.4203; Found:405.4195.

1,3-didodecyl imidazolium bromide - **1-Br**

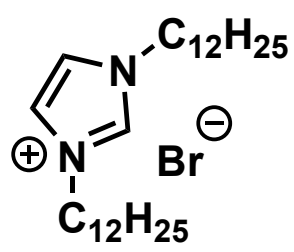

1-Br
485.64 g/mol
$C_{27}H_{53}N_2Br$

1,3-didodecyl imidazolium bromide (6.8 g, 14.0 mmol, 95%) was obtained by the reaction of imidazole (1 g, 14.7 mmol, 1 eq.), KOH (1.24 g, 22.1 mmol, 1.5 eq.), and 1-bromododecane (5.0 mL, 16.1 mmol, 1.1 eq. and 9.2 mL, 29.4 mmol, 2 eq.) in ACN.

^{1}H-NMR (500 MHz, $CDCl_3$) δ = 10.54 (s, 1 H), 7.36 (d, J = 1.6 Hz, 2 H), 4.34 (t, J = 7.4 Hz, 4 H), 1.93-1.87 (m, 4 H), 1.31-1.22 (broad, 36 H), 0.85 (t, J = 7.0 Hz, 6 H) ppm. Spectroscopic data match previously published data.[4]

m/z (ESI) Calcd. for $C_{27}H_{53}N_2Br$ [M-nBr, $C_{27}H_{53}N_2^+$]: 405.4203; Found: 405.4198.

1,3-didodecyl imidazolium iodine - **1-I**

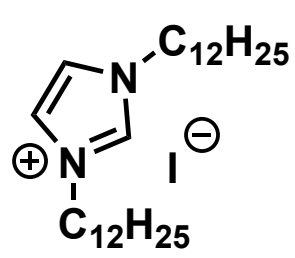

1-I
532.64 g/mol
$C_{27}H_{53}N_2I$

1,3-didodecyl imidazolium iodine (7.2 g, 13.5 mmol, 92%) was obtained by the reaction of imidazole (1 g, 14.7 mmol, 1 eq.), KOH (1.24 g, 22.1 mmol, 1.5 eq.), and 1-iodododecane (4 mL, 16.1 mmol, 1.1 eq. and 7.2 mL, 29.4 mmol, 2 eq.) in ACN.

^{1}H-NMR (500 MHz, $CDCl_3$) δ = 10.24 (s, 1 H), 7.39 (d, J = 1.6 Hz, 2 H), 4.34 (t, J = 7.4 Hz, 4 H), 1.94-1.89 (m, 4 H), 1.32-1.23 (broad, 36 H), 0.86 (t, J = 7.0 Hz, 6 H) ppm. Spectroscopic data match previously published data.[5]

m/z (ESI) Calcd. for $C_{27}H_{53}N_2I$ [M-nI, $C_{27}H_{53}N_2^+$]: 405.4203; Found: 405.4197.

1,3-didodecyl imidazolium tetraflouroborate - **1-BF_4**

1,3-didodecyl imidazolium tetrafluoroborate (0.466 g, 0.94 mmol, 92%) was obtained by the reaction of 1,3-didodecyl imidazolium bromide (0.500 g, 1.0 mmol, 1 eq.), KBF_4 (0.194 g, 1.5 mmol, 1.5 eq.) in acetone.

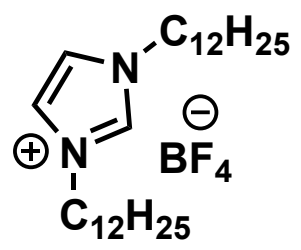

2-BF_4
441.19 g/mol
$C_{27}H_{53}N_2Cl$

^{1}H-NMR (500 MHz, $CDCl_3$) δ = 10.14 (s, 1 H), 7.29 (m, 2 H), 4.31 (t, J = 7.4 Hz, 4 H), 1.93-1.87 (m, 4 H), 1.32-1.24 (broad, 36 H), 0.87 (t, J = 7.0 Hz, 6 H) ppm. Spectroscopic data match previously published data.[6]

m/z (ESI) Calcd. for $C_{27}H_{53}N_2BF_4$ [M-nBF_4, $C_{27}H_{53}N_2^+$]: 405.4203; Found: 405.4199.

1,3-didodecyl benzimidazolium bromide **2**

1,3-didodecyl benzimidazolium bromide (4.9 g, 7.9 mmol, 97%) was obtained by the reaction of benzimidazole (1 g, 8.5 mmol, 1 eq.), KOH (0.7 g, 12.7 mmol, 1.5 eq.), and 1-bromododecane (2.9 mL, 9.3 mmol, 1.1 eq. and 5.2 mL, 16.9 mmol, 2 eq.) in ACN.

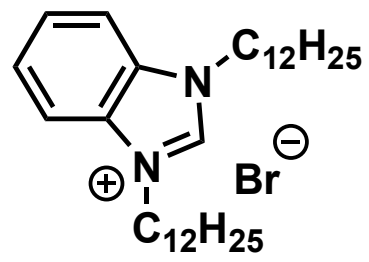

2-Br
535.70 g/mol
$C_{31}H_{55}N_2Br$

^{1}H-NMR (500 MHz, $CDCl_3$) δ = 11.43 (s, 1H), 7.72-7.63 (m, 4 H), 4.62 (t, J = 7.5 Hz, 4 H), 2.07-2.01 (m, 2 H), 1.43-1.22 (broad, 36 H), 0.86 (t, J = 7.0 Hz, 6 H) ppm. Spectroscopic data match previously published data.[4]

m/z (ESI) Calcd. for $C_{31}H_{55}N_2Br$ [M-nBr, $C_{31}H_{55}N_2^+$]: 455.4360; Found: 455.4353.

Procedure P2: Synthesis of imidazolium salts wingtip modifications

3,3'-(ethane-1,2-diyl)bis(1-dodecyl-1H-imidazol-3-ium) - **1-Biss**

3,3'-(ethane-1,2-diyl)bis(1-dodecyl-1H-imidazol-3-ium) was synthesized according to an adapted procedure from Lee et al.[7]

In short, Imidazole (29.3 mmol, 1 eq.) and KOH (1 eq.) were dissolved in absolute ACN, and 1-bromododecane (1 eq.) was added dropwise to the suspension. The resulting reaction mixture was refluxed for 24 h. After cooling the mixture to RT, the solvent was evaporated under reduced pressure. The resulting orange oil was used without further purification. The oil was dissolved in absolute toluene (10 mL), and Dibromoethane (0.6 eq.) was slowly added to the mixture at RT. The reaction mixture was then refluxed and stirred for 24 h. The precipitate was filtered off and washed with 2 x 10 mL toluene and 10 mL EtOAc. The resulting solid residue was dissolved in a minimal amount of MeOH, and the addition of diethyl ether precipitated the Bisimidazolium salt. Centrifugation of the suspension yielded in the white title compound.

3,3'-(ethane-1,2-diyl)bis(1-dodecyl-1H-imidazol-3-ium) (6.4 g, 9.7 mmol, 66 %) was obtained by the reaction of imidazole (2 g, 29.3 mmol, 1 eq.), KOH (1.6 g, 29.3 mmol, 1 eq) and 1-bromododecane (7.3 mL, 29.3 mmol, 1 eq) in ACN with subsequent reaction of the resulting oil with Dibromoethane (1.52 mL, 17.5 mmol, 0.6 eq) in toluene.

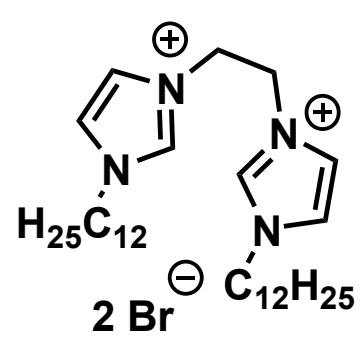

1-Biss
660.66 g/mol
$C_{32}H_{59}N_4Br_2$

^{1}H-NMR (500 MHz, $CDCl_3$) δ = δ 9.20 (s, 2H), 7.83 (s, 2H), 7.71 (s, 2H), 4.73 (s, 4H), 4.14 (t, J = 7.3 Hz, 4H), 1.74 (t, J = 7.4 Hz, 4H), 1.24 (m (br), 36H), 0.85 (t, J = 6.9 Hz, 6H) ppm. Spectroscopic data match previously published data.[7]

m/z (ESI) Calcd. for $C_{32}H_{60}N_4Br_2$ [M-2Br, $C_{32}H_{60}N_4^{2+}$]: 250.2404; Found: 250.2402.

Procedure P3: Synthesis of Imidazolinium Salt

Imidazolinium salt was synthesized according to an adapted procedure from Seidl et al.[8] and Malecki et al.[9]

In short, ethylenediamine (3 mmol, 1 eq.) was dissolved in CH_2Cl_2 (10 mL), and dodecanal (2 eq.) was added slowly. The resulting solution was stirred at RT for 1 h, and solid sodium triacetoxyborohydride (2.3 eq.) was added portion-wise to the reaction mixture. The mixture was stirred at RT for 24 h, quenched with saturated $KHCO_3$ (pH 9), and extracted with CH_2Cl_2 (3 x 50 mL). The organic phases were concentrated and acidified with methanolic HCl (0.5 M), and diethyl ether was added. The resulting precipitate was collected, dissolved in CH_2Cl_2, and alkalized with saturated $KHCO_3$ (pH 9). The aqueous phase was extracted with additional CH_2Cl_2. Finally, the combined organic phases were dried over $MgSO_4$, and drying in vacuo yielded the diamine intermediate N1,N2-didodecylethane-1,2-diamine. The intermediate was analyzed by NMR and dissolved in dioxane (5 mL), followed by the addition of triethyl orthoformate (3 eq.) and ammonium bromide (1 eq.). The mixture was heated to 100 °C for 24 h, concentrated under reduced pressure, and the addition of diethyl ether yielded the solid title compound.

N1,N2-didodecylethane-1,2-diamine

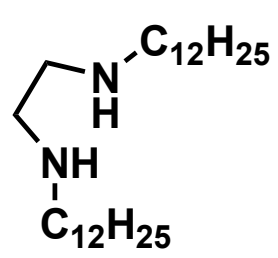

396.74 g/mol
$C_{26}H_{56}N_2$

N1,N2-didodecylethane-1,2-diamine (1.1 g, 2.7 mmol, 97%) was obtained by the reaction of Ethylendiamine (200 µL, 3.0 mmol, 1 eq.), Dodecanal (1.3 mL, 6.0 mmol, 2 eq.), and triacetoxyborohydride (1.5 g, 6.9 mmol, 2.3 eq.) in CH_2Cl_2.

^{1}H-NMR (500 MHz, $CDCl_3$) δ = 2.79 (s, 4 H), 2.63 (t, J = 8.3 Hz, 4 H), 1.57-1.46 (m, 4 H), 1.36-1.18 (broad, 36 H), 0.86 (t, J = 6.9 Hz, 6 H) ppm. Spectroscopic data match previously published data.[8]

1,3-didodecyl imidazolinium bromide – **3-Br**

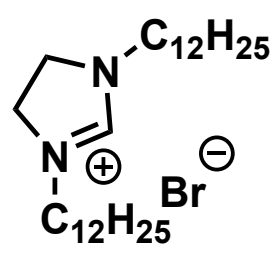

3-Br
487.65 g/mol
$C_{27}H_{55}N_2Br$

1,3-didodecyl imidazolinium bromide (0.96 g, 2.0 mmol, 73 %) was obtained by the reaction of N1,N2-didodecylethane-1,2-diamine (1.1 g, 2.7 mmol, 1 eq.), triethyl orthoformate (1.34 mL, 8.1 mmol, 3 eq.), and ammonium bromide (264 mg, 2.7 mmol, 1 eq.).

^{1}H-NMR (500 MHz, $CDCl_3$) δ = 9.61 (s, 1 H), 3.96 (s, 4 H), 3.62 (dd, J = 7.6 Hz, 4 H), 1.65 (p, J = 7.3 Hz, 4 H), 1.32-1.22 (broad, 36 H), 0.86 (t, J = 6.9 Hz, 6 H) ppm. Spectroscopic data match previously published data.[9]

m/z (ESI) Calcd. for $C_{27}H_{55}N_2Br$ [M-nBr, $C_{27}H_{55}N_2^+$]: 407.4360; Found: 407.4355.

Procedure P4: Synthesis of 1,2,3-Triazoles

Triazoles were synthesized according to an adapted procedure by Rivara et al.[10]

In a 20 mL vial, the used solvent mix was degassed, and bromine (3 mmol, 1 eq.) and sodium azide (1.05 eq.) were added and stirred at RT for 10 min. $CuSO_4$•5 H_2O (5 mol%) followed by sodium L-ascorbate (50 mol%) was added and the mixture was stirred for an additional 5 min at RT. Finally, the alkyne (1.05 eq.) was added, and the mixture was heated to 70 °C for 16 h. The reaction was cooled to RT, and NH_4OH (6 mL, 25 %) was added, and the resulting mixture was extracted with DCM (4 x 15 mL). Subsequent washing with brine (20 mL) and water (2 x 20 mL) resulted in the crude product. The crude product was dried in vacuo and used without further purification.

1,4-didodecyl-1H-1,2,3-triazole

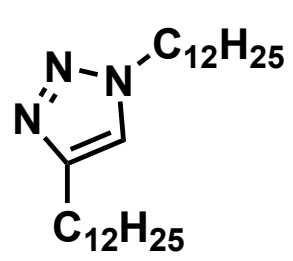

405.71 g/mol
$C_{26}H_{50}N_3$

1,4-didodecyl-1H-1,2,3-triazole (0.931 g, 2.295 mmol, 76 %) was obtained by reacting 1-dodecyl bromide (0.72 mL, 3 mmol, 1 eq.), sodium azide (0.205 g, 3.150 mmol, 1.05 eq.), 1- tetradecyne (0.77 mL, 3.150 mmol, 1.05 eq.), $CuSO_4$•5 H_2O (0.037 g, 0.150 mmol, 5 mol%), and sodium L-ascorbate (0.297 g, 1.500 mmol, 0.5 eq.) in DMF/H_2O (2:1, 6 mL).

^{1}H-NMR (500 MHz, $CDCl_3$) δ = 7.25 (s, 1H), 4.30 (t, J = 7.1 Hz, 2 H), 2.70 (t, J = 7.5 Hz, 2 H), 1.89-1.86 (m, 2 H), 1.67 (m, 2 H), 1.30-1.25 (broad, 38 H), 0.87 (t, J = 6.9 Hz, 6 H) ppm. Spectroscopic data match previously published data.[11]

m/z (ESI) Calcd. for $C_{26}H_{51}N_3$ [M+nH, $C_{26}H_{51}N_3^+$]: 406.4156; Found: 406.4153.

Procedure P6: Synthesis of 1,2,3-Triazolium Salts

Triazolium salts were synthesized according to an adapted procedure by Liang et al.[12]

Crude 1,2,3-triazoles (1 mmol, 1 eq.) were dissolved in ACN, and ethyl halide (5 eq.) was added. The resulting mixture was heated to 90 °C for 48 h. The reaction mixture was concentrated under reduced pressure, and the crude triazolium salt was obtained by the addition of cold diethyl ether and subsequent collection of solids by centrifugation.

1-dodecyl-3-ethyl-4-undecyl-1H-1,2,3-triazol-3-ium Bromide - **4-Br**

1,4-didodecyl-1H-1,2,3-triazole (0.406 g, 1.000 mmol, 1 eq.) was reacted with 1-bromoethane (0.15 mL, 2.029 mmol, 5 eq.) in ACN. Workup yielded 1,4-didodecyl-2-ethyl-1H-1,2,3-triazol-2-ium (0.437 g, 0.850 mmol, 85 %) as a white crystalline solid.

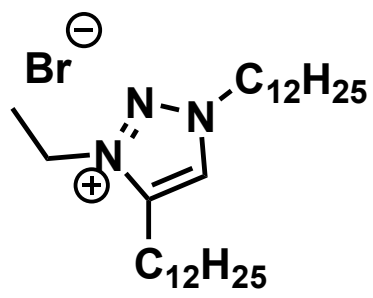

4-Br
514.88 g/mol
$C_{28}H_{56}N_3Br$

^{1}H-NMR (600 MHz, $CDCl_3$) δ = 9.59 (s, 1H), 4.79 (t, J = 7.4 Hz, 2 H), 4.53 (q, J = 7.3 Hz, 2 H), 2.88-2.86 (m, 2 H), 2.04-2.00(m, 2 H), 1.80-1.74 (m, 2 H), 1.64 (t, J = 7.3 HZ, 3H), 1.42-1.37 (m, 2 H), 1.36-1.22 (broad, 36 H), 0.85 (t, J = 6.8 Hz, 6 H) ppm.
^{13}C-NMR (600 MHz, $CDCl_3$) δ = 143.8, 130.2, 54.4, 46.7, 32.0, 29.7, 29.7, 29.64, 29.6, 29.5, 29.4, 29.4, 29.3, 29.2, 29.0, 27.8, 26.3, 23.7, 22.8, 14.4, 14.2 ppm. Full NMR analysis is depicted in Figure S19.

m/z (ESI) Calcd. for $C_{28}H_{56}N_3Br$ [M-nBr, $C_{28}H_{56}N_3^+$]: 434.4469; Found: 434.4473.

Procedure P7: Synthesis of DDS@AuNPs

DDS@AuNPs were synthesized by adaptation of a protocol by Chechik and coworkers under ambient conditions.[13]

In short, $HAuCl_4*3H_2O$ (0.40 mol, 136 mg, 1 eq.) and tetraocylammonium bromide (0.41 mol, 132 mg, 1 eq.) were combined and dissolved in 40 mL of water. After adding 16 mL of toluene, the heterogeneous mixture was stirred vigorously for 15 minutes. The aqueous phase was discarded using a separation funnel. Didodecylsulfide (1.13 mol, 494 mg, 3 eq.) was dissolved in 24 mL toluene and added to the collected organic phase. In a separate vessel, $NaBH_4$ (0.8 mmol, 30.4 mg, 2 eq.) was dissolved in 16 mL of water. The aqueous solution was added in one portion to the stirring organic phase at room temperature. Over the course of 20 minutes, the organic phase changed color from yellow to brown and finally red. After phase separation, the organic phase was dried over $MgSO_4$ and the solvent removed under reduced pressure. The resulting nanomaterial was precipitated using 500 mL of Acetone and collected by centrifugation. Complete drying and redispersing of the DDS@AuNPs resulted in narrowly dispersed nanoparticles, which were used in the following reactions.

Procedure P8: Synthesis of NHC@AuNPs, NHC@AgNPs and NHC@CuNPs

In a 5 mL vial, the NHC precursor (20.6 mmol, 1 eq.), $KHCO_3$ (22.7 mmol, 1.1 eq.) and 900 µL 1,4-dioxane were combined and stirred at 25 °C for 10 min at 600 rpm. Subsequently, 100 µL of a DDS@AuNP, DDS@AgNPs or OYA@CuNPs dispersion in toluene (10 mg/mL) was added, and the reaction mixture was stirred for 24 h. The reaction was worked up under ambient conditions. Gold nanoparticles were precipitated by the addition of 40 mL technical-grade acetone, followed by centrifugation to collect the precipitate. The solid residue was redispersed in n-hexane, and the washing step was repeated three times to remove any excess ligand. After the final redispersion, the nanoparticles were filtered through a 20 µm pore-size PTFE syringe filter to remove residual base. The resulting dispersion was stored under ambient conditions, where the particles remained stable for over 5 weeks.

All ligand exchange reactions were performed according to P1 with distinct variations of ligand, temperature, base, inert condition, and equivalents, which are stated in the respective experiment. Complete ligand exchange was still observed after upscaling the optimized reaction up to 10 mg of DDS@AuNPs using **1-Br**.

Procedure P9: Synthesis of DDS@AgNPs

DDS@AuNPs were synthesized by adaptation of a protocol by Padmos and coworkers under ambient conditions.[14]

In short, silveracetate (0.05 mol, 8.3 mg, 1 eq.), didodecylsulfide (0.05 mol, 18.5 mg, 1 eq.) and tetraocylammonium bromide (0.15 mol, 81.9 mg, 3 eq.) were combined and dissolved in 25 mL of toluene. The mixture was sonicated and formed a clear solution. In a separate vessel, $NaBH_4$ (0.5 mmol, 18.9 mg, 10 eq.) was dissolved in 20 mL of water. The aqueous solution was added in one portion to the stirring organic phase at room temperature. Over the course of 30 minutes, the organic phase

changed color from colorless to red then yellow and finally dark yellow. After phase separation, the organic phase was dried over $MgSO_4$ and the solvent removed under reduced pressure. The resulting nanomaterial were redispersed in toluene and filtered through a 0.2 µm teflon syringe filter and precipitated using 20 mL of technical grade ethanol and collected by centrifugation. Complete drying and redispersing of the DDS@AgNPs resulted in narrowly dispersed nanoparticles, which were used in the following reactions.

Procedure P10: Synthesis of OYA@CuNPs

OYA@CuNPs were synthesized by adaption of a protocol by Aissa and coworkers.[15] It is to note that small copper nanoparticles are notoriously sensitive to air and moisture and should be treated exclusively in a glovebox or strict Schlenk conditions. All further modifications reported in this work were handled under strict inert conditions.

$CuCl(PPh_3)_3$ (0.26 mol, 23.2 mg, 1 eq.) was dissolved in 2.5 mL of toluene brought to 100 °C after which oleyl amine (0.16 mol, 52 µL, 0.4 eq) is added. In a separate vial tert-butylammonium borane complex (0.24 mol, 21.7 mg, 1 eq.) is dissolved in 1.5 mL toluene and heated to 100 °C. After both solutions have reached the reaction temperature vial B is added to vial A and the mixture is stirred for 15 min. The solvent of red dispersion is removed under reduced pressure under inert conditions. The remaining black oil is then redissolved in a minimal amount of THF and the particles precipitated with an excess (~10:1) of dry ACN. The precipitate is collected using a centrifuge at 6K rcf over 3 minutes. The resulting redispersible solid yields the desired **OYA@CuNPs**. It is to note that these particles are only shelf stable by the addition of octadecene during the reaction. Since we immediately used the particles for ligand exchange experiments no octadecene was added.

Procedure P11: Synthesis of bulk NHC@Au

Gold films with a thickness of 80 nm were deposited on a 2 cm^2 glass by physical vapour deposition using a Kurt J. Lesker PVD75 PROLine at a deposition rate of 0.5 Å/s. To enhance the adhesion of the gold layer to the glass slide, a 4 nm thick chromium (Cr) adhesion-layer was deposited. The slides were then cleaned via 3 sonication cycles per solvent with water, acetone, and ethanol for 30 minutes. After cleaning, all substrates were dried at 70 °C. Surface functionalization was performed by fully immersing the gold-coated substrate in a 10 mM solution of 1-Br (1 eq) with $KHCO_3$ (1.1 eq) in dioxane at 25 °C without stirring. The substrates were allowed to incubate for 24 h, followed by the same washing protocol as before to ensure washing all unbound imidazolium species. The functionalized glass slide was then dried and analysed using XPS.

Analysis of the Nanoparticles with changing reaction conditions

Full analysis of DDS@AuNPs and NHC@AuNPs synthesised using optimized reaction conditions

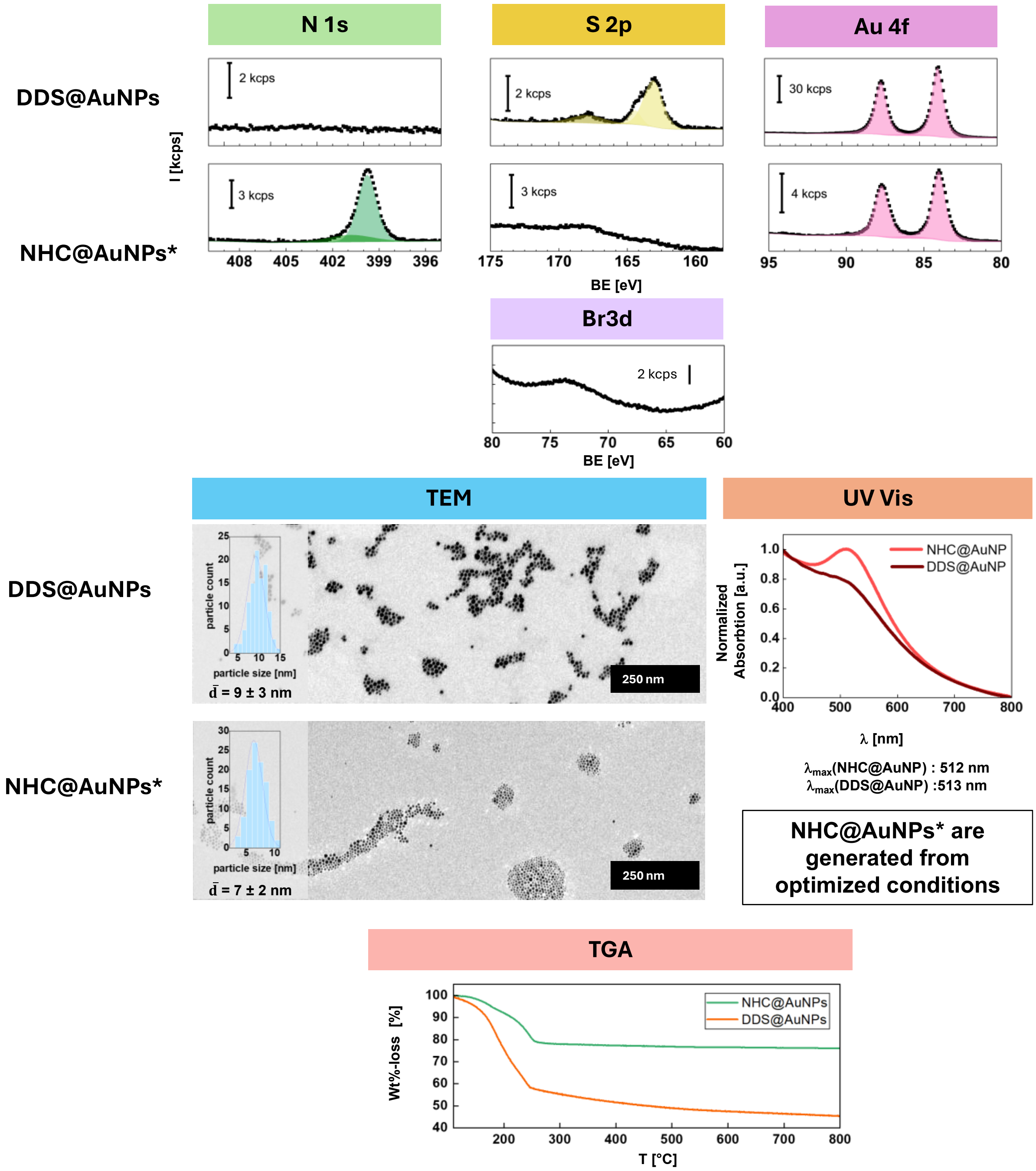

Figure S1. Comparison of DDS@AuNPs with NHC@AuNPs by N 1s, S 2p, Au 4f high resolution XPS-spectra, TEM Micrographs, TGA analysis, and UV-Vis spectra. Additionally Br3d spectra of the NHC functionalized particles are shown to provide further information about the surface capping.

**NHC@AuNPs shown yielded from optimized exchange conditions using 1,4-dioxane as solvent for 24 h at 25 °C and approximately 10 equivalents of exchange ligand with $KHCO_3$ as base.*

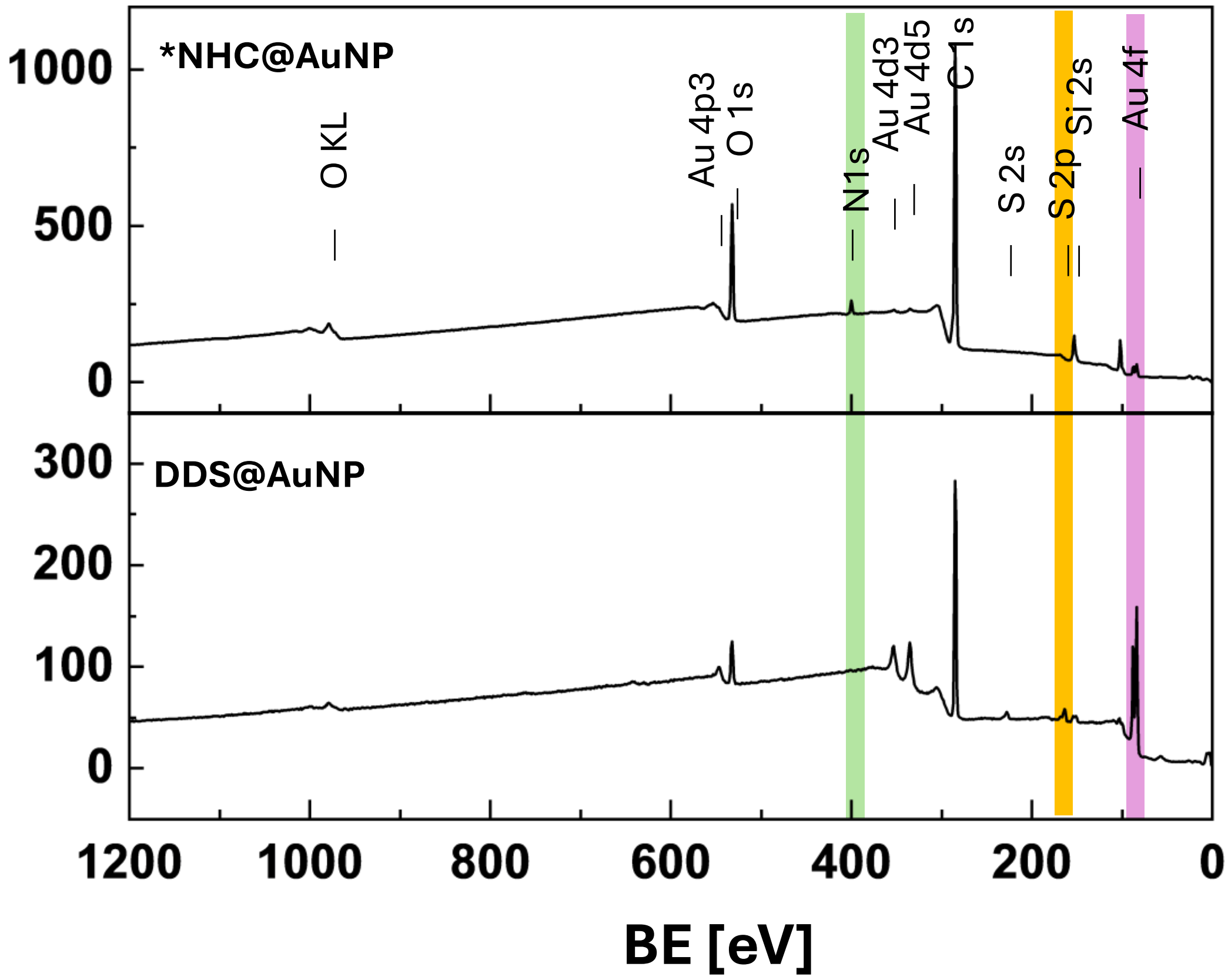

Figure S2. Comparison of XPS survey spectra of DDS@AuNPs with NHC@AuNPs.

**NHC@AuNPs yielded from optimized exchange conditions using the 1,4-dioxane as solvent for 24 h at 25 °C and 13 equivalents of exchange ligand with $KHCO_3$ as base.*

qNMR analysis of the optimized nanoparticles

For the quantification of the surface-bound NHC ligands by quantitative ^{1}H NMR (qNMR), 3 mg of NHC@AuNPs obtained under the optimized reaction conditions were dispersed in dioxane, and 100 mg of KI was added in a single portion. The reaction mixture was stirred vigorously at 60 °C until the characteristic red plasmonic color had completely disappeared and a homogeneous yellow solution was obtained, indicating complete digestion of the nanoparticles. The reaction mixture was subsequently filtered, and all volatiles were removed under reduced pressure. The resulting yellow residue was redissolved in 250 µL of $CDCl_3$ and transferred to an NMR tube. Quantitative transfer of all reaction products was ensured by rinsing the reaction vessel twice with 125 µL portions of $CDCl_3$, which were combined with the initial solution.
A preliminary ^{1}H-NMR spectrum was acquired to estimate the appropriate amount of internal standard required for quantitative analysis. Subsequently, 250 µL of a 9 mM trimethoxybenzene stock solution was added to the NMR tube and mixed thoroughly. The final solution was analyzed on a 700 MHz NMR spectrometer using 64 scans to maximize the signal-to-noise ratio.
Owing to the complex chemistry accompanying nanoparticle digestion, multiple resonances attributable to ligand-derived species were observed in the aromatic region. Therefore, the entire spectral region between 8.2 and 6.2 ppm, corresponding to the NHC backbone resonances, was integrated and referenced against the trimethoxybenzene singlet at 6.09 ppm for quantification.

The NHC coverage was calculated using the following formulas:

Used internal standard:

$$n_{TriBenz} = c_{TriBenz} * V_{TriBenz} = 0.9\ mM * 0.25\ mL = 0.002\ mmol$$

Normalization of the integrated protons

$$I_n(TriBenz) = \frac{I(TriBenz)}{n_{\frac{Protons}{Molecule}}} = \frac{2.1}{3} = 1.42$$

$$I_n(NHC) = \frac{I(NHC)}{n_{\frac{Protons}{Molecule}}} = \frac{3.04}{2} = 1.52$$

$$I_{ratio} = \frac{I_n(NHC)}{I_n(TriBenz)} = 1.06$$

Calculation of the n and m of NHCs

$$n(\ NHC) = I_{ratio} * n(TriBenz) = 1.06 * 0.002\ mmol = 0.0023\ mmol$$

$$m = n(NHC) * M(NHC) = 0.0023 * 407\frac{g}{mol} = 0.96\ mg\ \leftrightarrow 32\ wt\%$$

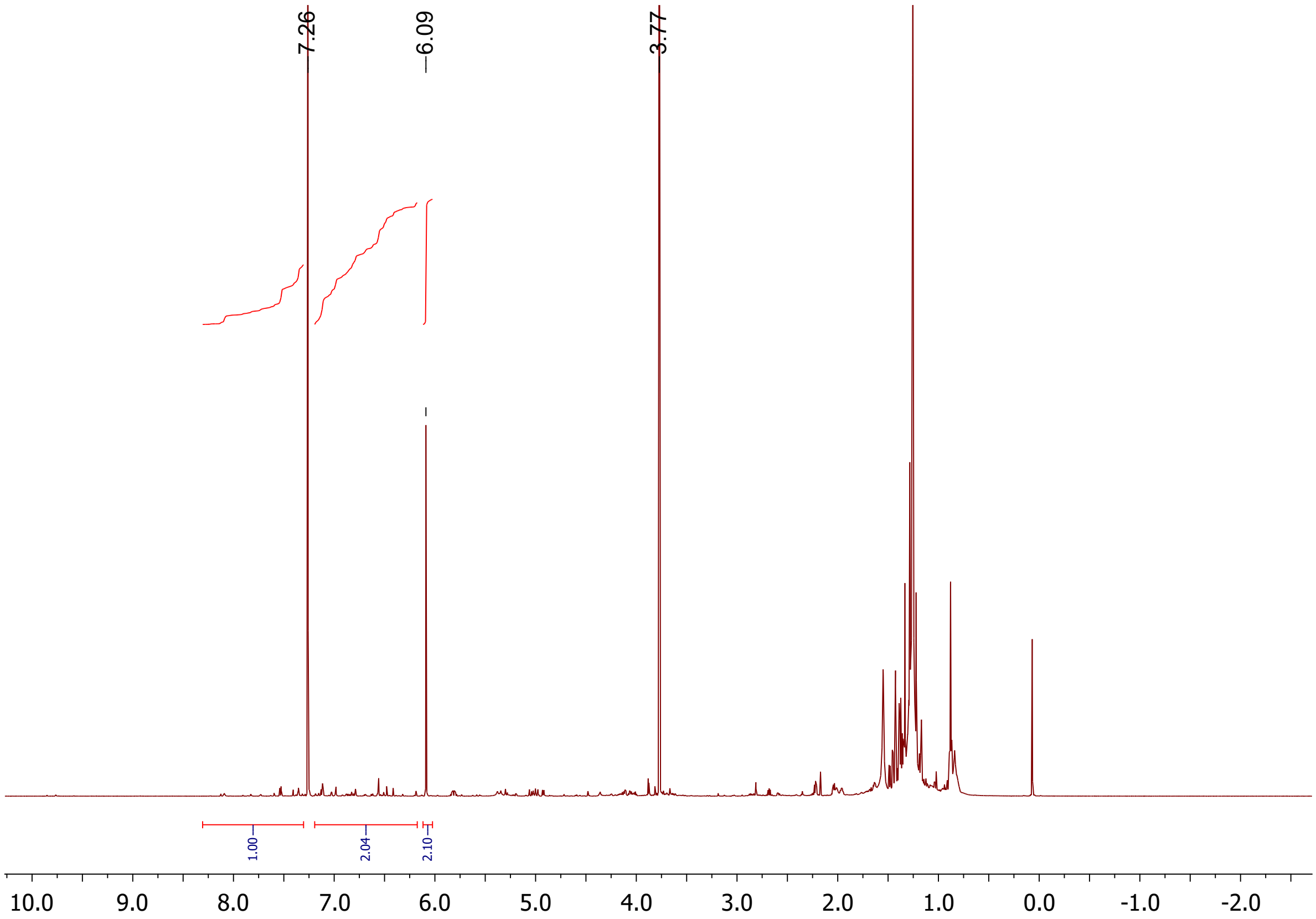

Figure S3. Quantitative NMR investigation of the digested particles using potassium iodine. 2,4,6-Trimethoxybenzene was used for the complete digestion.

Analysis of the ligand exchange reaction using 1-Br with variation of solvent

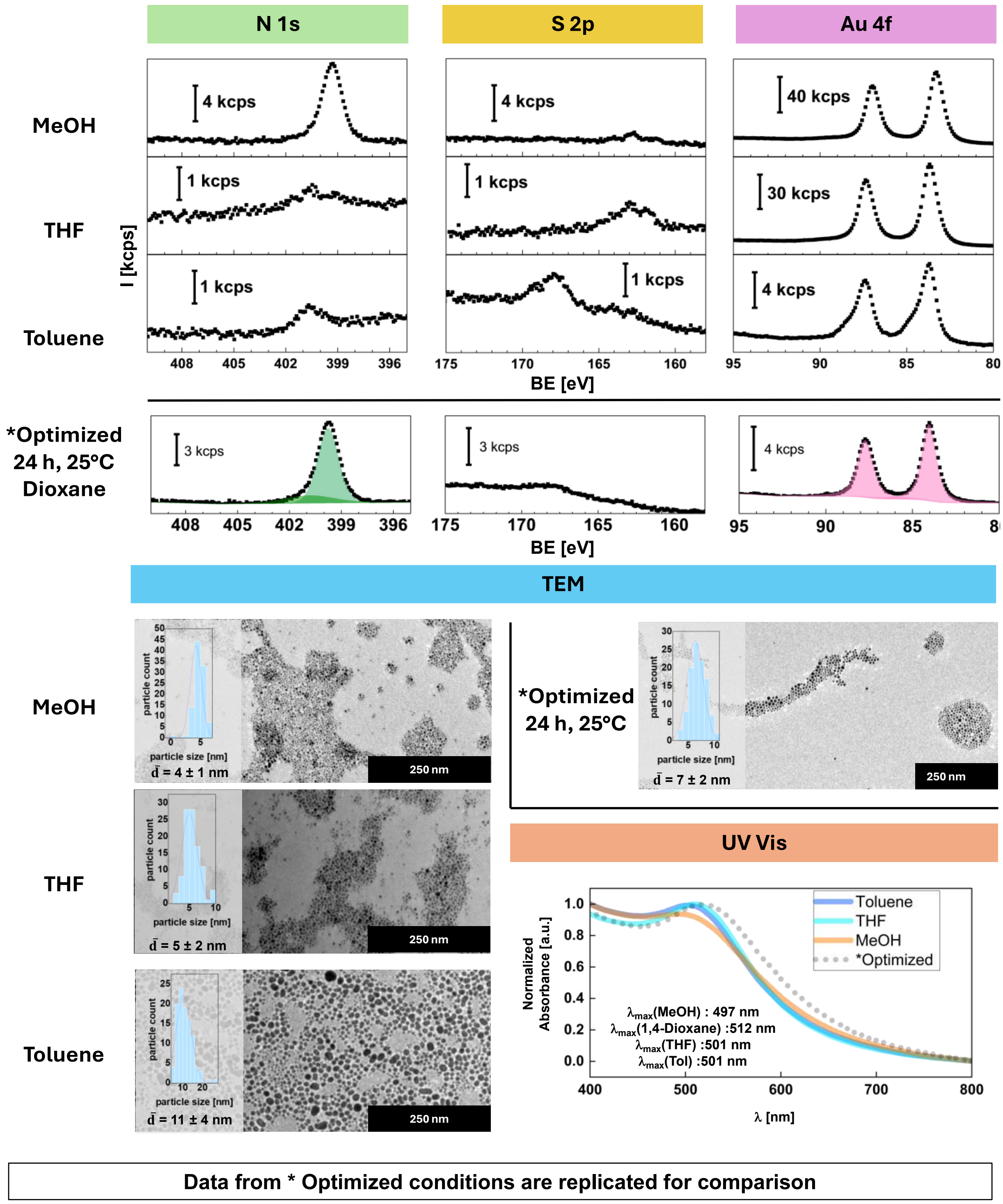

Figure S4. N 1s, S 2p, Au 4f high-resolution XPS spectra, TEM Micrographs, and UV-Vis spectra of NHC@AuNPs after the ligand exchange using the following reaction conditions. Variation of solvents at 40 °C using 13 equivalents of exchange ligand and $KHCO_3$ as base. Fully optimized conditions* are also replicated in this figure for comparison.

Analysis of the particles with variation of reaction time at 40 °C

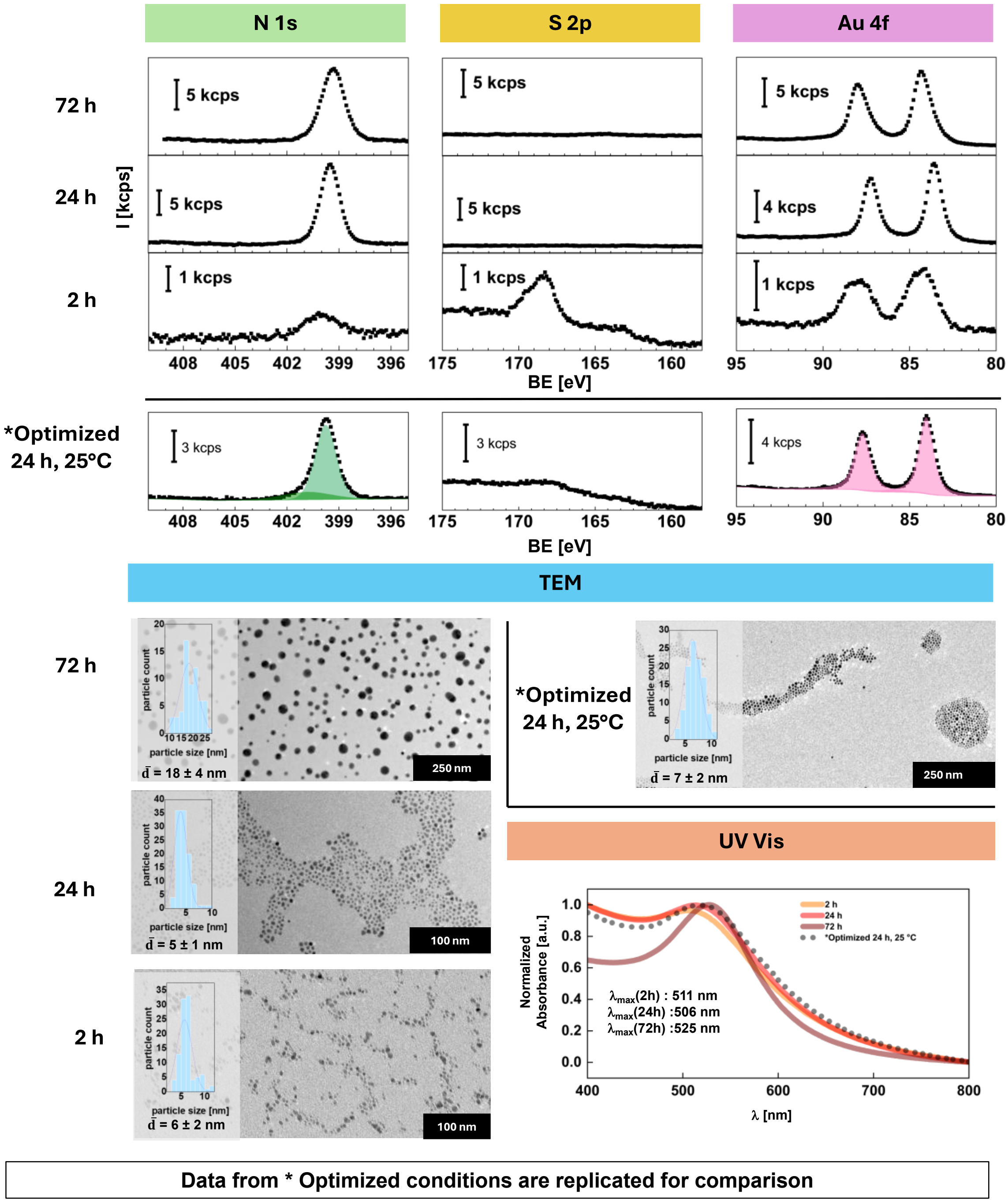

Figure S5. N 1s, S 2p, Au 4f high-resolution XPS spectra, TEM Micrographs, and UV-Vis spectra of NHC@AuNPs after the ligand exchange using the following reaction conditions. Variation of reaction time at 40 °C using 1,4-dioxane as solvent and 13 equivalents of exchange ligand using $KHCO_3$ as base. Fully optimized conditions* are also replicated in this figure for comparison.

Analysis of the ligand exchange using 1-Br with variation of reaction time at 25 °C

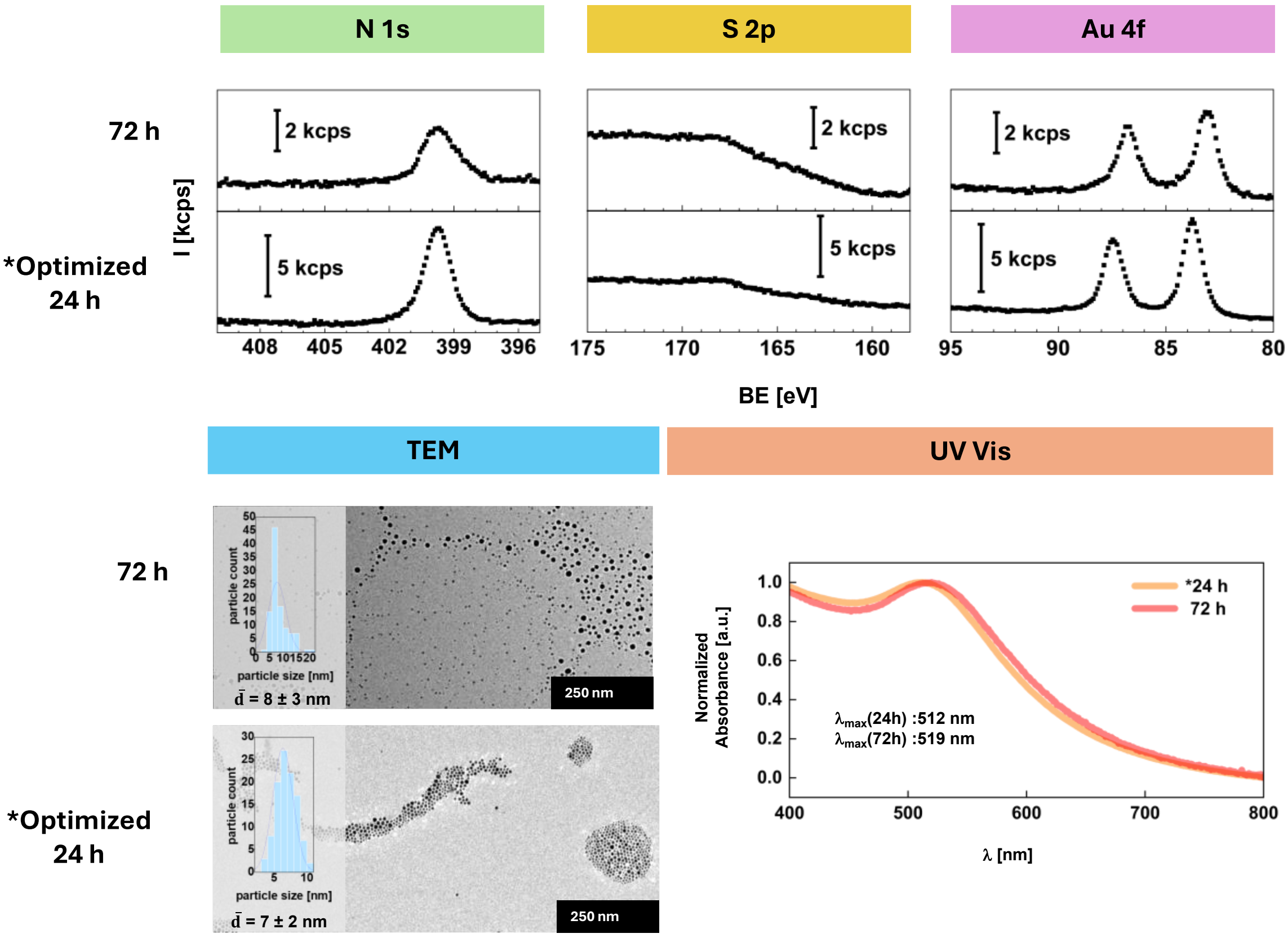

Figure S6. N 1s, S 2p, Au 4f high-resolution XPS spectra, TEM Micrographs, and UV-Vis spectra of NHC@AuNPs after the ligand exchange using the following reaction conditions. Variation of reaction time at 25 °C using 1,4-dioxane as a solvent with 13 equivalents of exchange ligand using $KHCO_3$ as base. *The best-performing condition in the screening is indicated by an asterisk.

Analysis of the ligand exchange using 1-Br with variation of reaction temperature

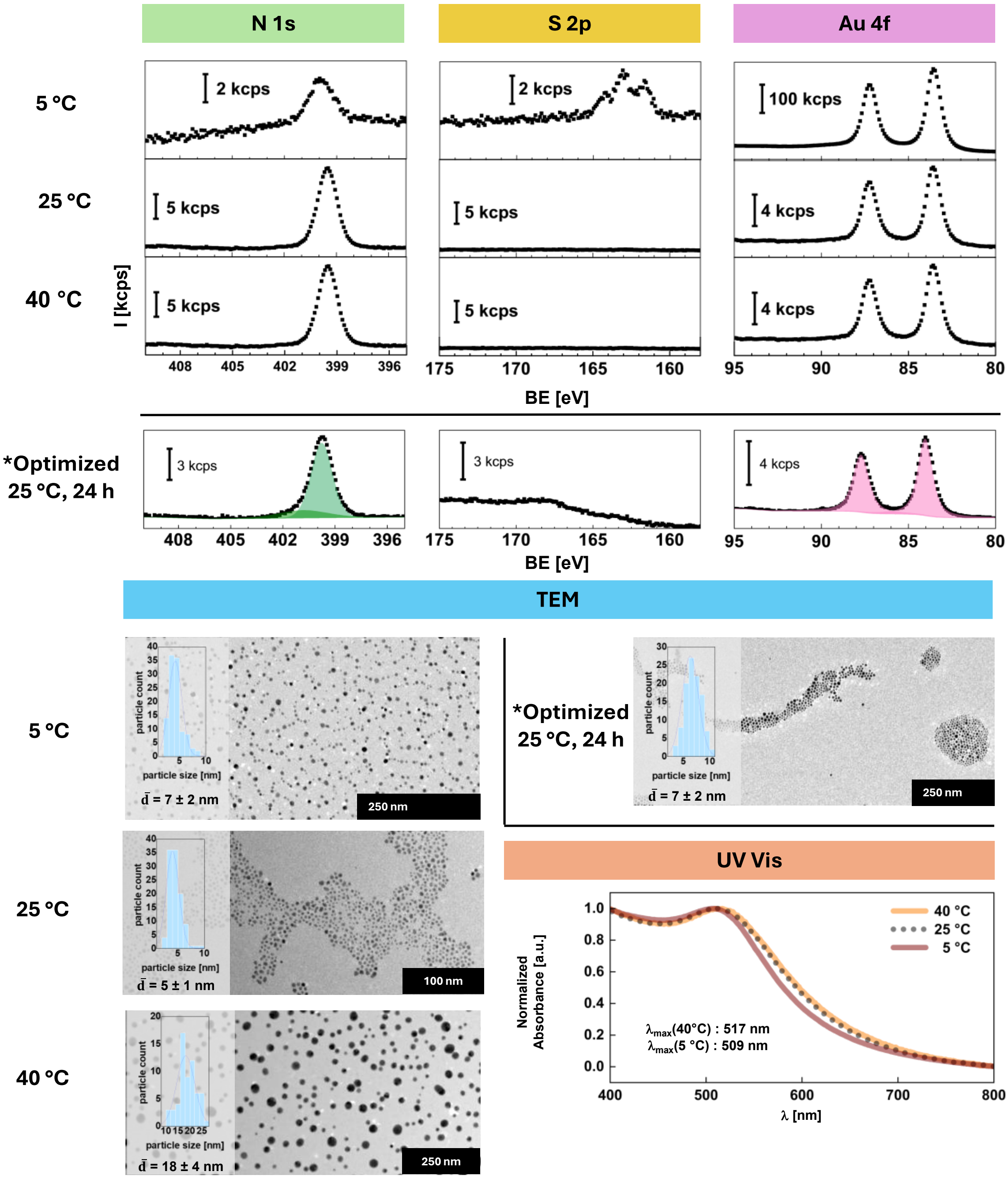

Data from 25°C, 40°C and * Optimized conditions are replicated for comparison

Figure S7. N 1s, S 2p, Au 4f high-resolution XPS spectra, TEM Micrographs, and UV-Vis spectra of NHC@AuNPs after the ligand exchange using the following reaction conditions. Variation of reaction temperature over 72 h using 1,4-dioxane as a solvent with 13 equivalents of exchange ligand using $KHCO_3$ as a base. Fully optimized conditions* and results in dioxane at 40 °C are also replicated in this figure for comparison.

Analysis of the ligand exchange using 1-Br with variation of the employed equivalent of NHC precursor

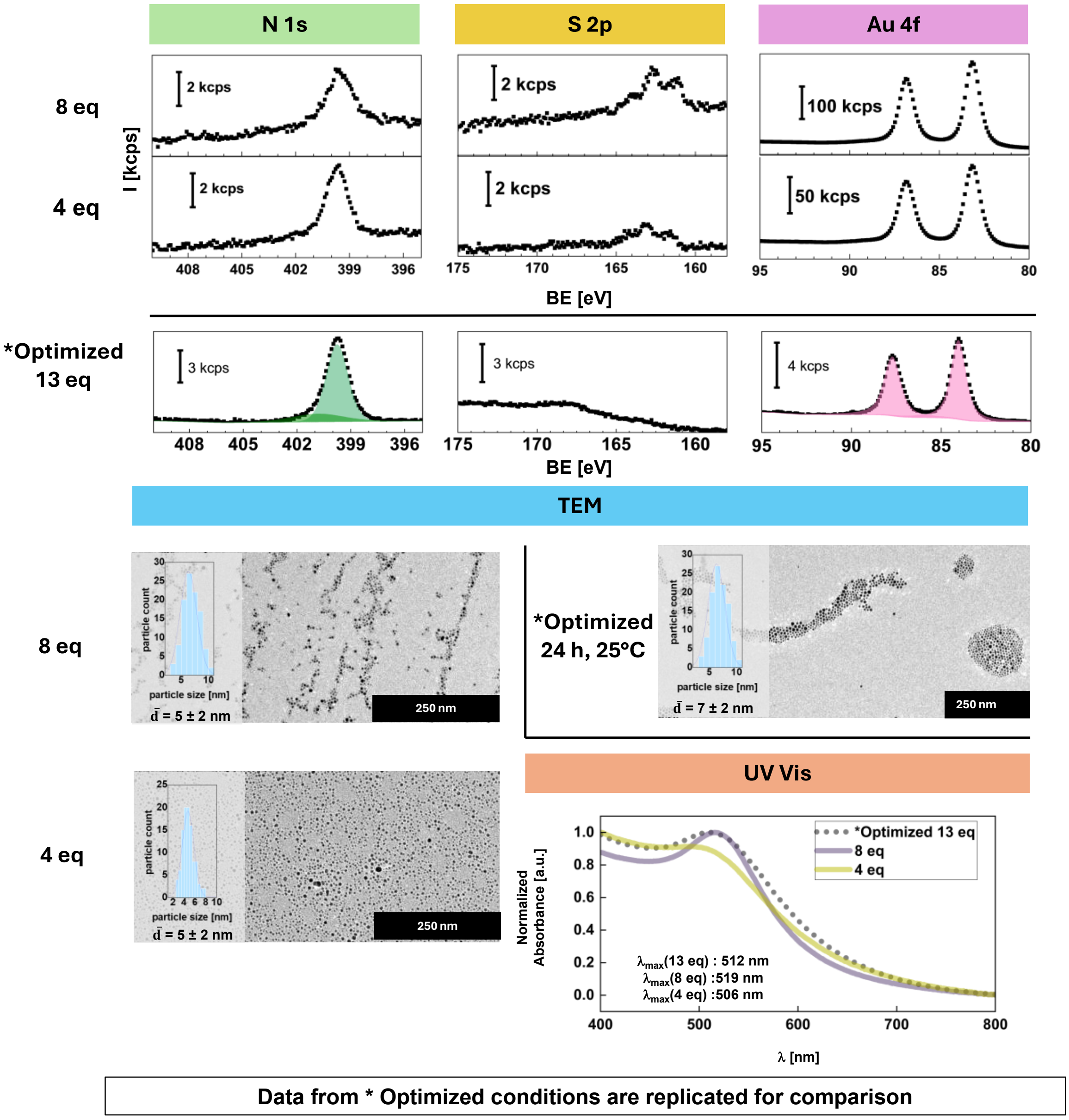

Figure S8. N 1s, S 2p, Au 4f high-resolution XPS spectra, TEM Micrographs, and UV-Vis spectra of NHC@AuNPs after the ligand exchange using the following reaction conditions. Optimized conditions at 25 °C, in 1,4-dioxane using $KHCO_3$ as base with changing equivalents of exchange ligands. Fully optimized conditions* are also replicated in this figure for comparison.

Analysis of the ligand exchange using 1-Br with variation of the employed base

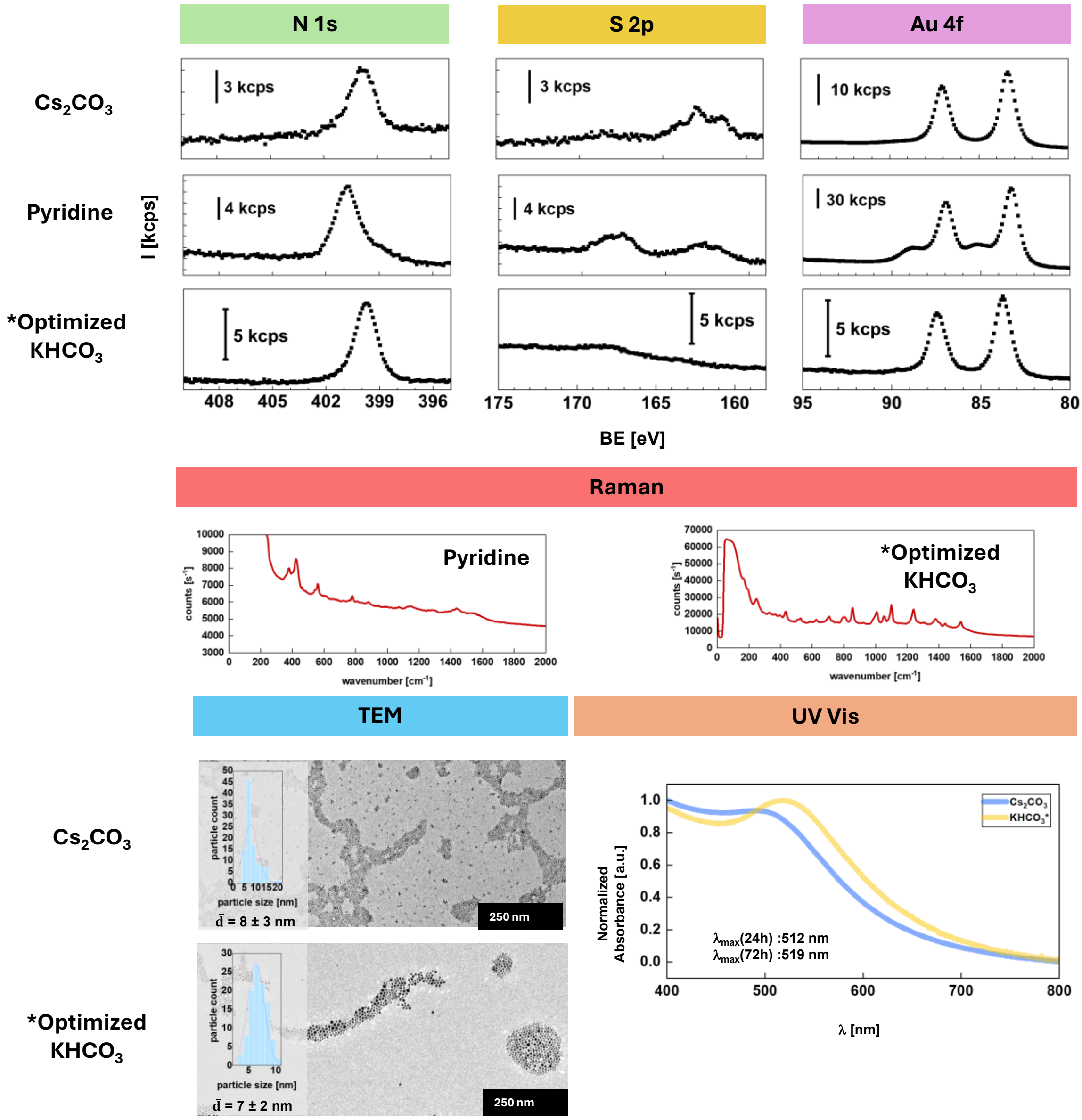

Figure S9. N 1s, S 2p, Au 4f high-resolution XPS spectra, TEM Micrographs, Raman Spectroscopy and UV-Vis spectra of NHC@AuNPs after the ligand exchange using the following reaction conditions. Optimized conditions at 25 °C, in 1,4-dioxane using different bases. Fully optimized conditions* are also replicated in this figure for comparison.

Stability of the Surface Plasmon of NHC*@AuNPs achieved by optimized conditions under relevant conditions

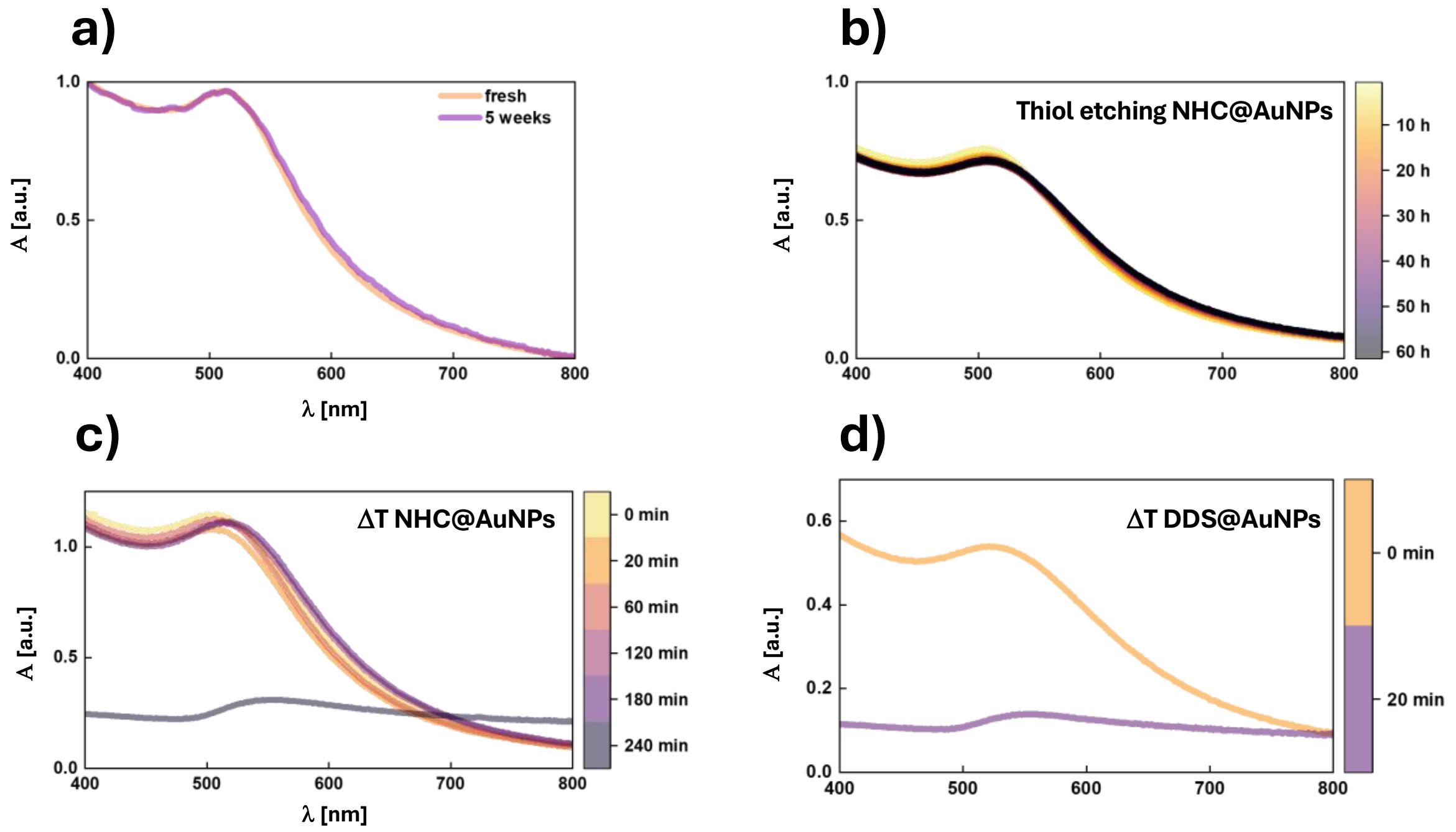

Figure S10. Stability of the respective nanomaterial under relevant conditions. a) Long time stability under ambient conditions storage. b) Stability in 5 mM Dodecanthiol at rt. Resistance under elevated temperature (100 °C) and air atmosphere unfor c) NHC*@AuNPs and d) DDS@AuNPs.

Probing the robustness of the one-pot ligand exchange

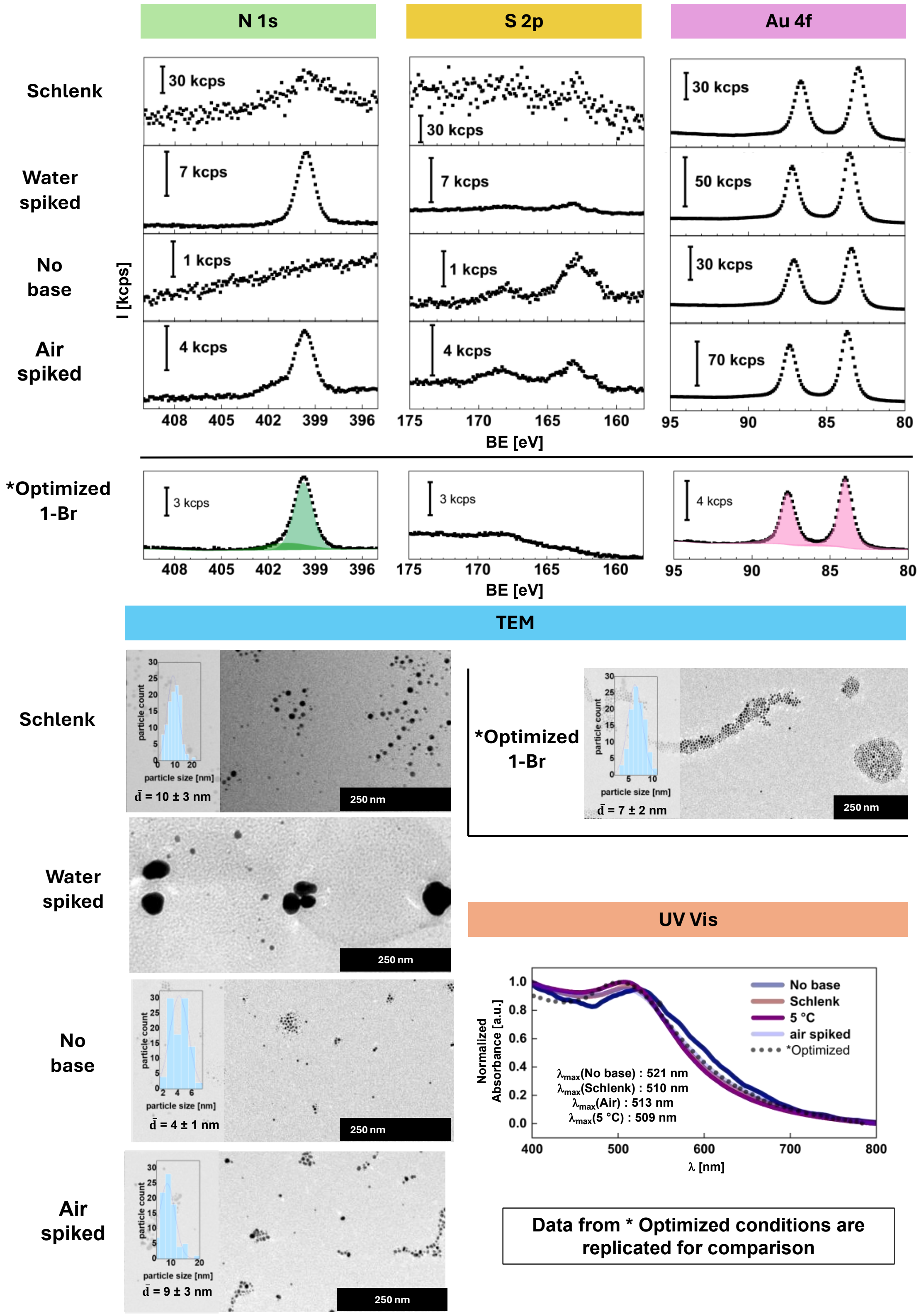

Figure S11. N 1s, S 2p, Au 4f high-resolution XPS spectra, TEM Micrographs, and UV-Vis spectra of NHC@AuNPs after the ligand exchange using the following reaction conditions. Optimized conditions at 25 °C, 13 equivalents of exchange ligand in 1,4-dioxane, $KHCO_3$ was used as base. Investigation of the robustness of the reaction under an air-argon mix, with the addition of water, under Schlenk conditions, and without a mild base. Fully optimized conditions* are also replicated in this figure for comparison.

Analysis of the ligand exchange using 1-X with variation of counter ion on the NHC precursor

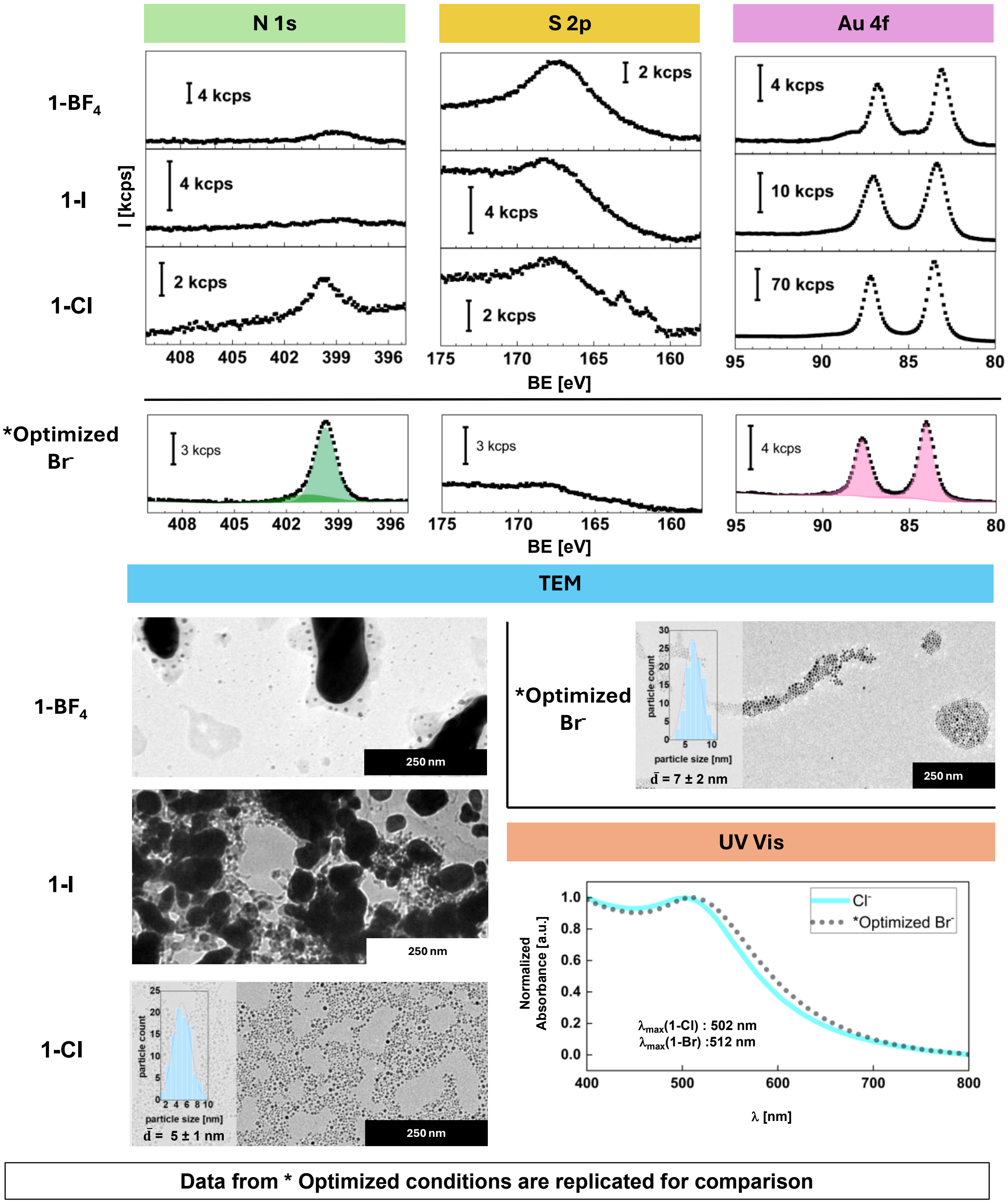

Figure S12. N 1s, S 2p, Au 4f high-resolution XPS spectra, TEM Micrographs, and UV-Vis spectra of NHC@AuNPs after the ligand exchange using the following reaction conditions. Optimized conditions were used to screen different NHC precursor-salts at 25 °C for 24 h using 13 equivalents of exchange ligand in 1,4-dioxane with $KHCO_3$ as base. Due to aggregation UV-Vis spectra and size distribution of 1-I and 1-BF_4 are not shown. Fully optimized conditions* are also replicated in this figure for comparison.

Stability of the Surface Plasmon of DDS@AuNPs in dioxane for 24 h with the addition of KCl, KBr, KI and KBF_4 via UV-Vis

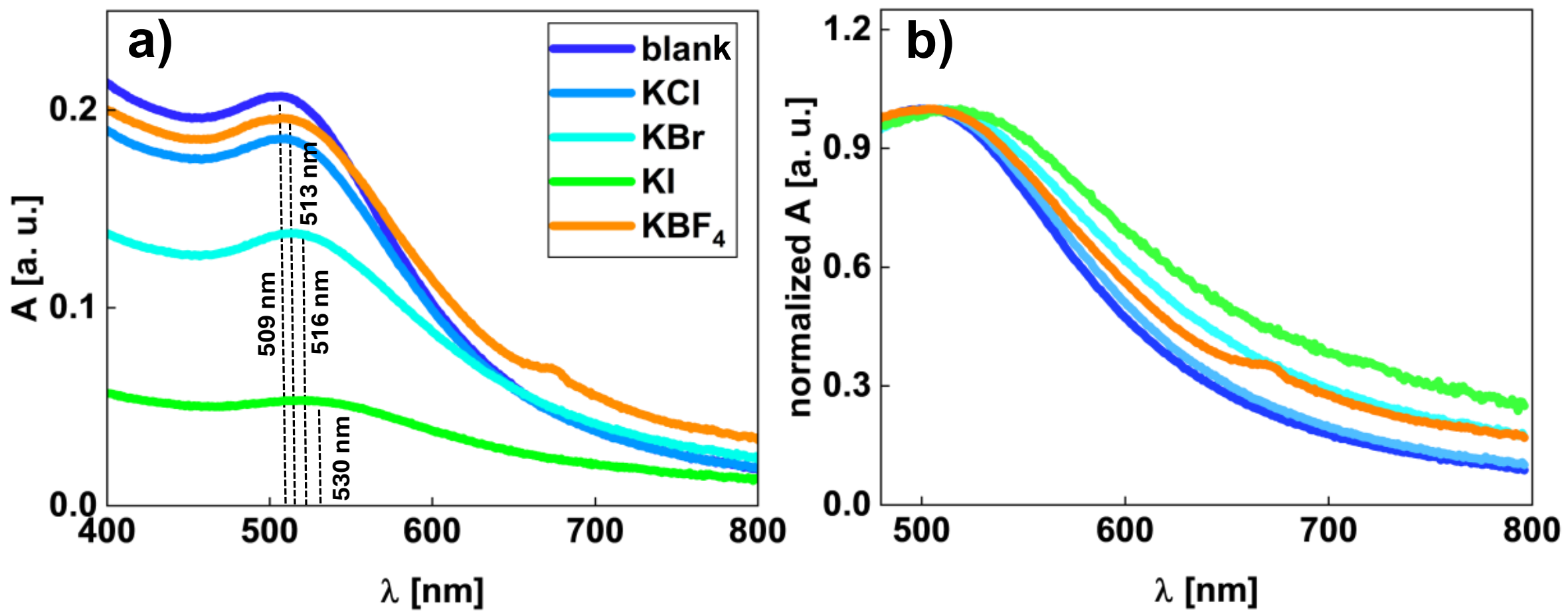

Figure S13. UV-Vis spectra of particles after stirring in dioxane for 24 h at 25 °C under inert conditions with the addition of different salts. Comparison of **a)** the redshifts of LSPR maxima and **b)** FWHP in the normalized spectra of DDS@AuNPs without the addition of salts show clear influence of the counter ions.

Analysis of the ligand exchange using optimized conditions with different NHC and MIC precursors

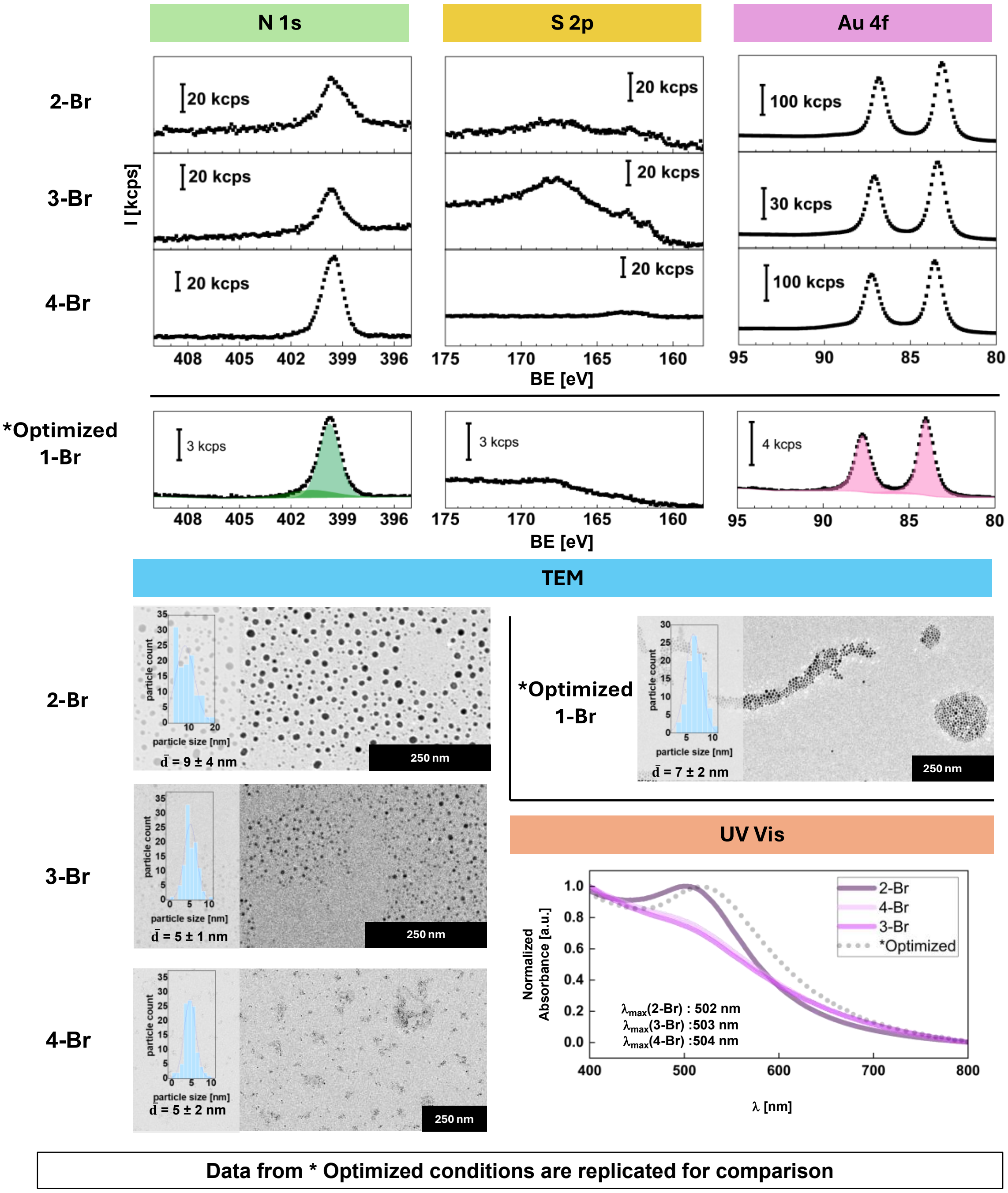

Figure S14. N 1s, S 2p, Au 4f high-resolution XPS spectra, TEM Micrographs, and UV-Vis spectra of NHC@AuNPs and MIC@AuNPs after the ligand exchange using the following reaction conditions. Optimized conditions at 25 °C with approximately 10 equivalents of exchange ligand in 1,4-dioxane with $KHCO_3$ as base were used to screen different types of NHCs and MIC. Fully optimized conditions* using **1-Br** are also replicated in this figure for comparison.

Full Raman spectra of synthesized NHC/MIC@AuNPs

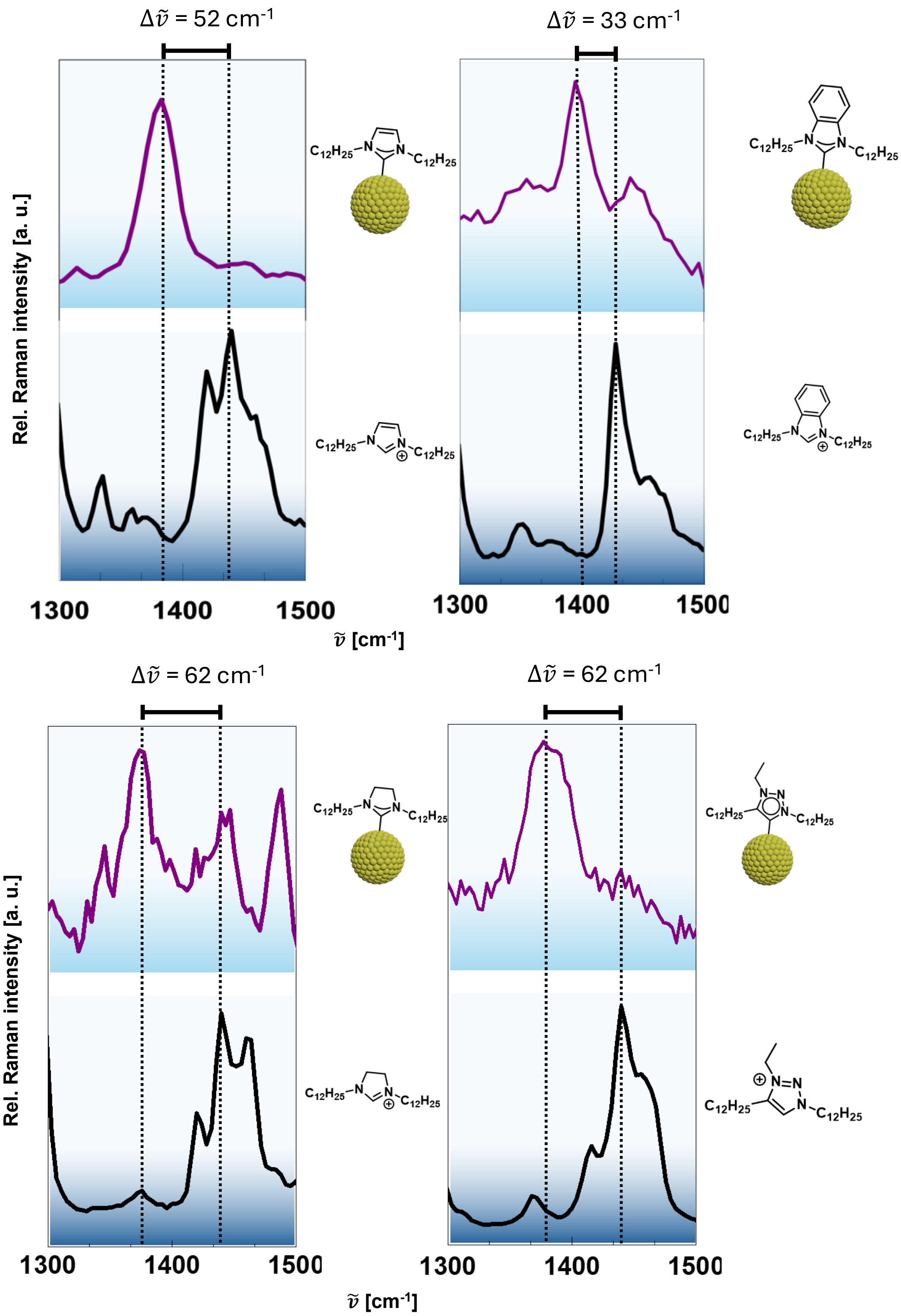

Figure S15. Analysis of the NHC/MIC gold surface interaction using surface-enhanced Raman spectroscopy. The observed redshift of the N-C stretch band of the NHC/MIC-Precursor compared with the NHC/MIC on the surface in Raman spectroscopy confirms the successful formation of a C-Au bond. All spectra shown here are raw spectra to ensure no baseline correction inflicts the observed shift.

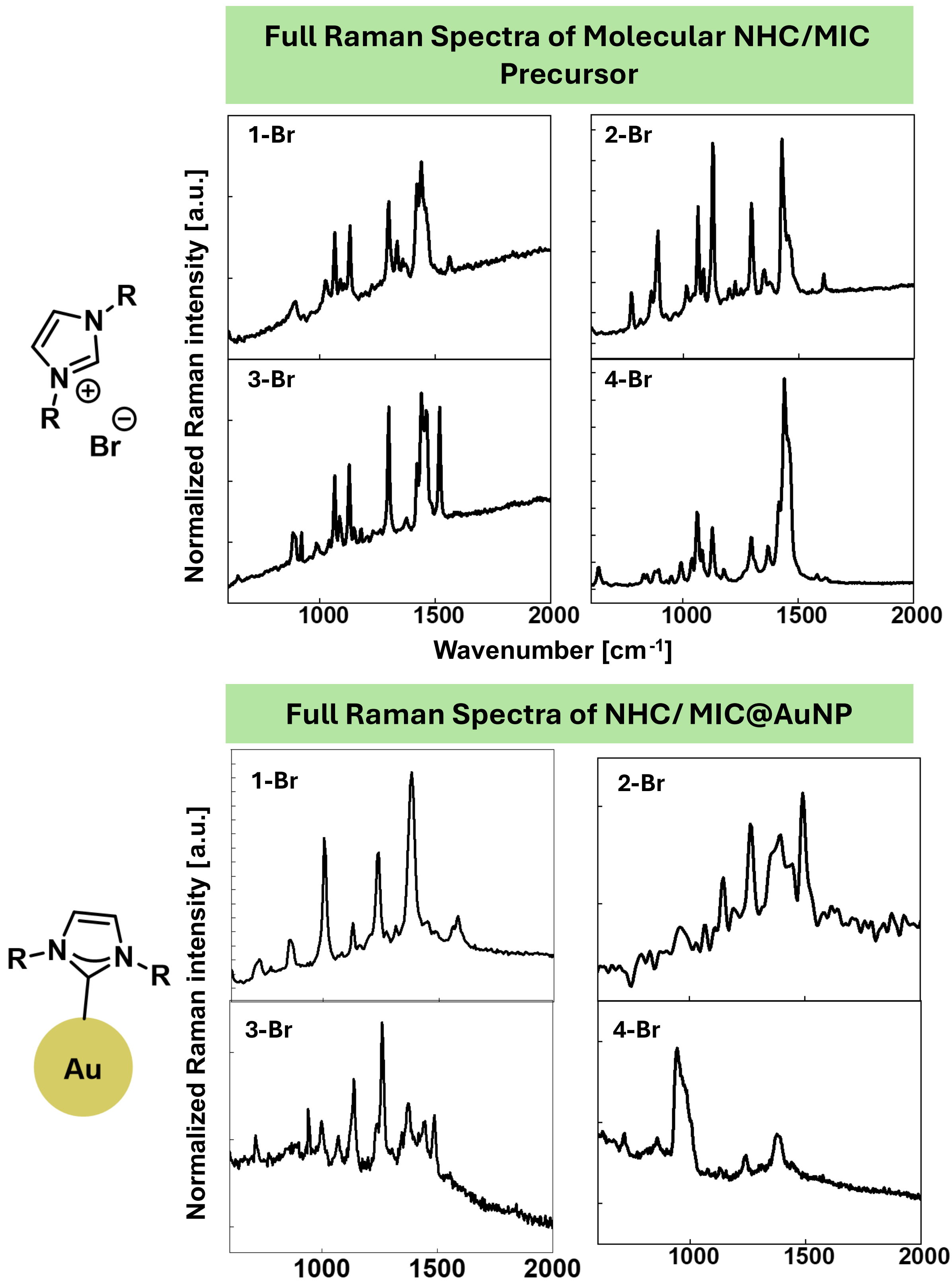

Figure S16. Complete Raman spectra of the NHC/MIC precursor and SERS measurements of the NHC/MIC functionalized AuNPs.

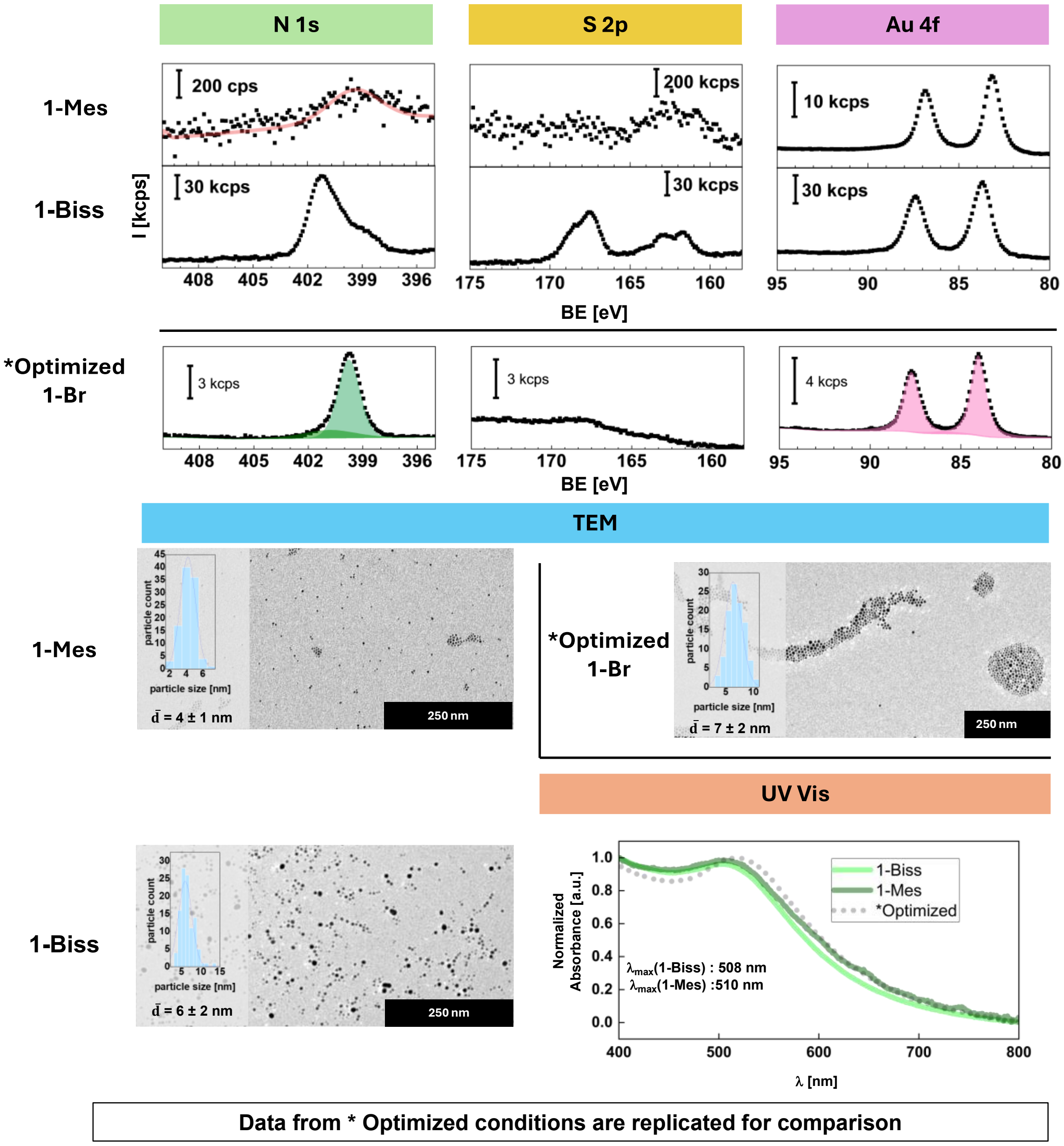

Figure S17. N 1s, S 2p, Au 4f high-resolution XPS spectra, TEM Micrographs, and UV-Vis spectra of NHC@AuNPs with different sidechain modifications after the ligand exchange using the following reaction conditions. Optimized conditions at 25 °C with 13 equivalents of exchange ligand in 1,4-dioxane with KHCO3 as base were used to screen different wingtip modifications of classical NHCs. Fully optimized conditions* using **1-Br** are also replicated in this figure for comparison.

Full analysis of optimized reaction conditions on different starting materials

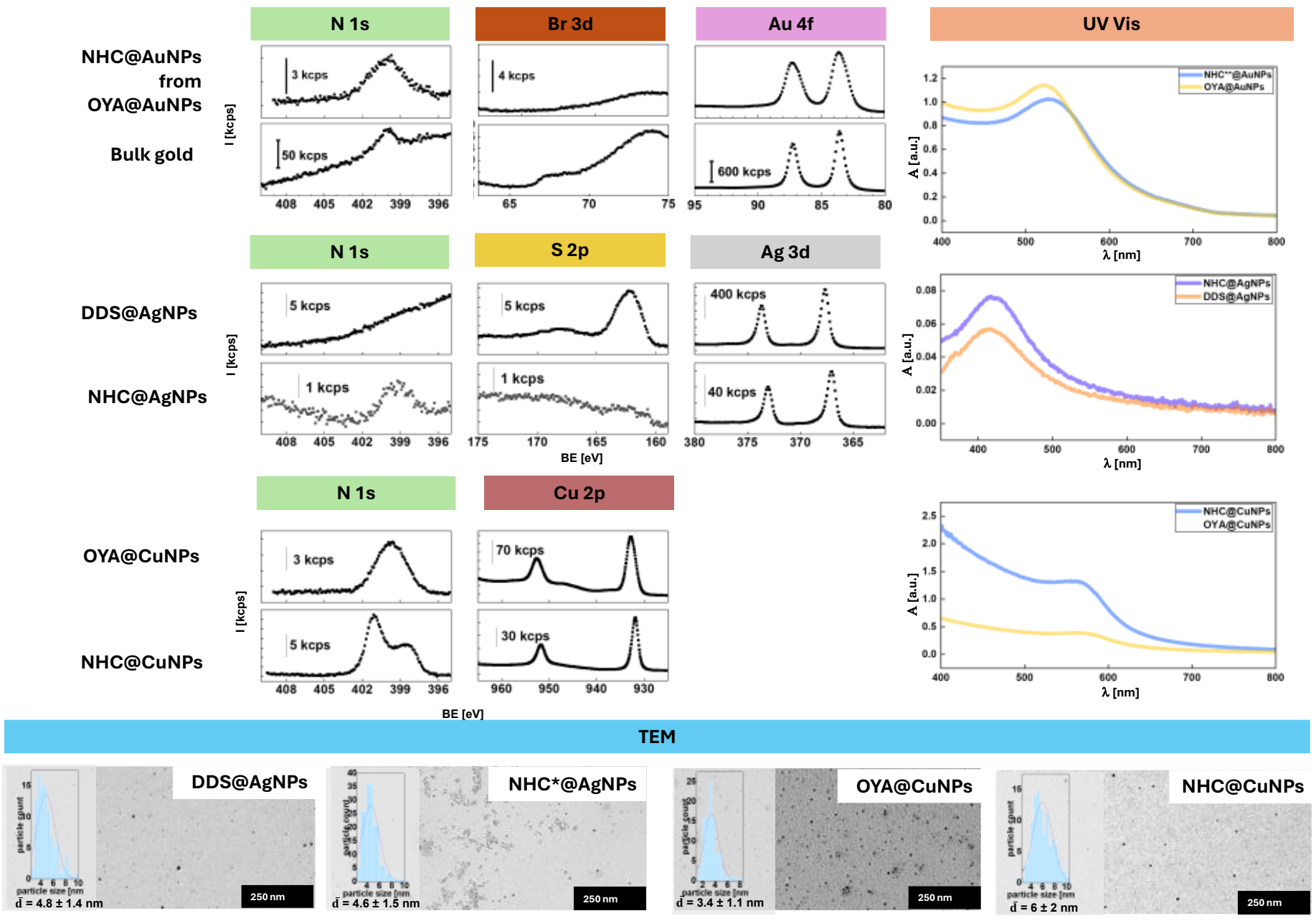

Figure S18. N 1s, Br 3d, Au 4f high resolution XPS-spectra, NHC@Au after the ligand exchange using the following reaction conditions. Optimized conditions at 25 °C in 1,4-dioxane with $KHCO_3$ as base were used. The glass slide with freshly PVD-deposited gold film was immersed in the reaction mixture.

Surface enhanced Raman Analysis of optimized reaction conditions on OYA@AuNPs, DDS@AgNPs and OYA@CuNPs

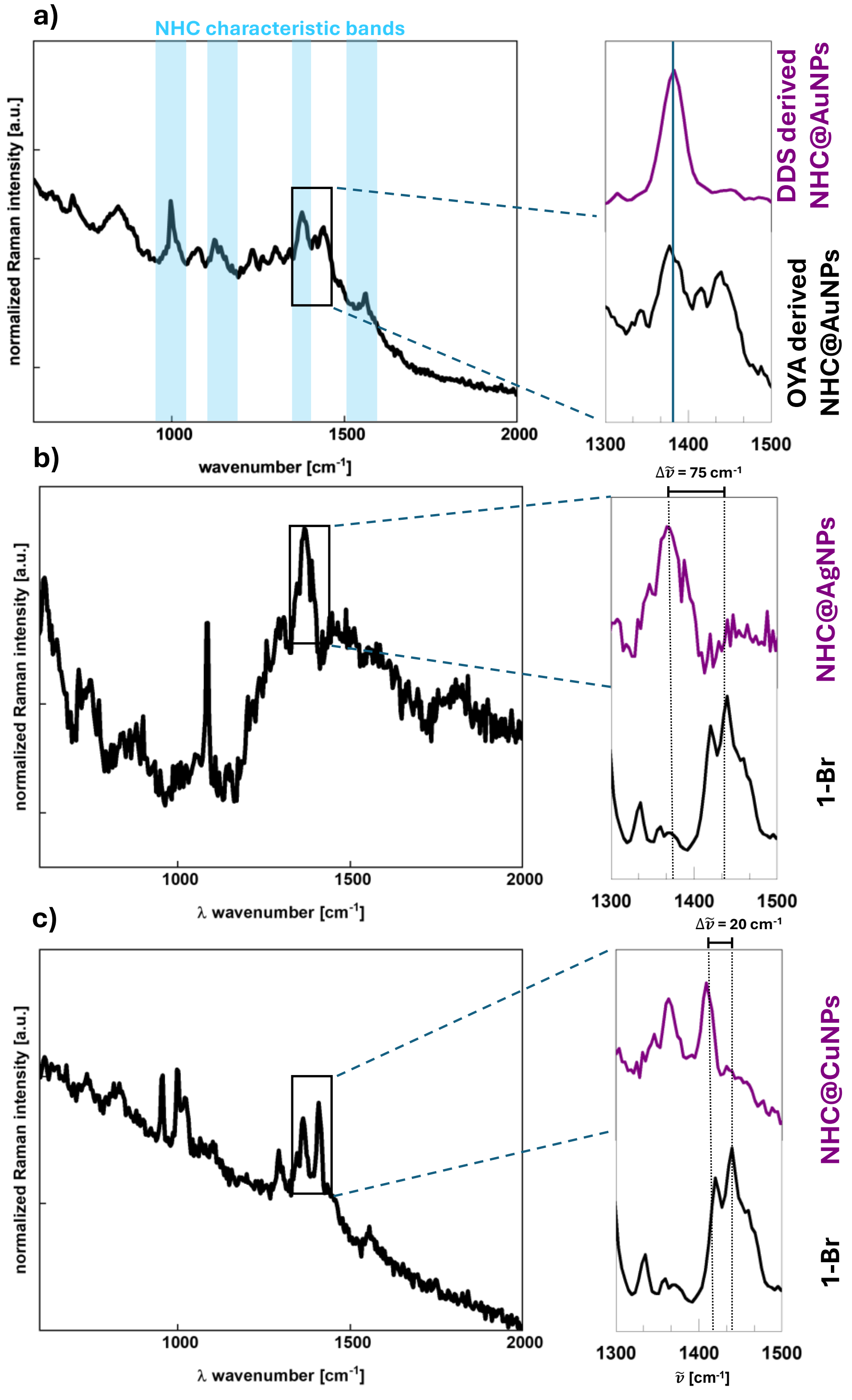

Figure S19. SERS analysis of NHC@AuNPs derived from of OYA@AuNPs, DDS@AgNPs and OYA@CuNPs. Comparison of the characteristic NCN band with NHC@AuNPs from DDS@AuNPs verifies the successful implementation of NHC on the OYA@AuNPs nanoparticles surface. For DDS@AgNPs and OYA@CuNPs comparison with characteristic spectrum of the ligand showed a significant redshift.

Time resolved comparison of ^{1}H-NMR spectra of the 1-Br after $KHCO_3$ treatment

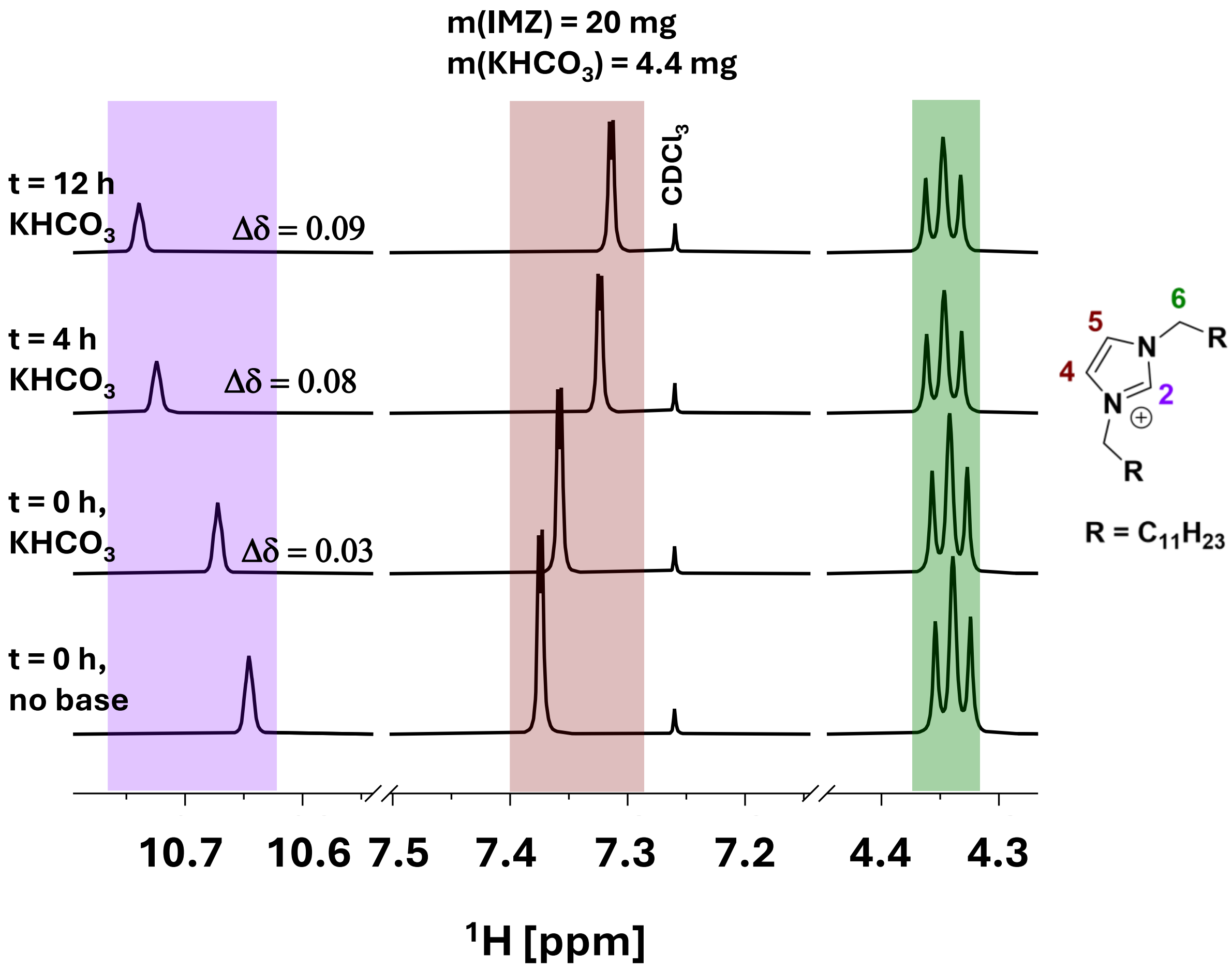

Figure S20. Comparison of ^{1}H-NMR spectra of the **1-Br** before and after treatment with $KHCO_3$ at room temperature in 1,4-dioxane for 0, 4 and 12 h.

Comparison of ^{1}H-NMR spectra of the respective NHC precursors after $KHCO_3$ treatment

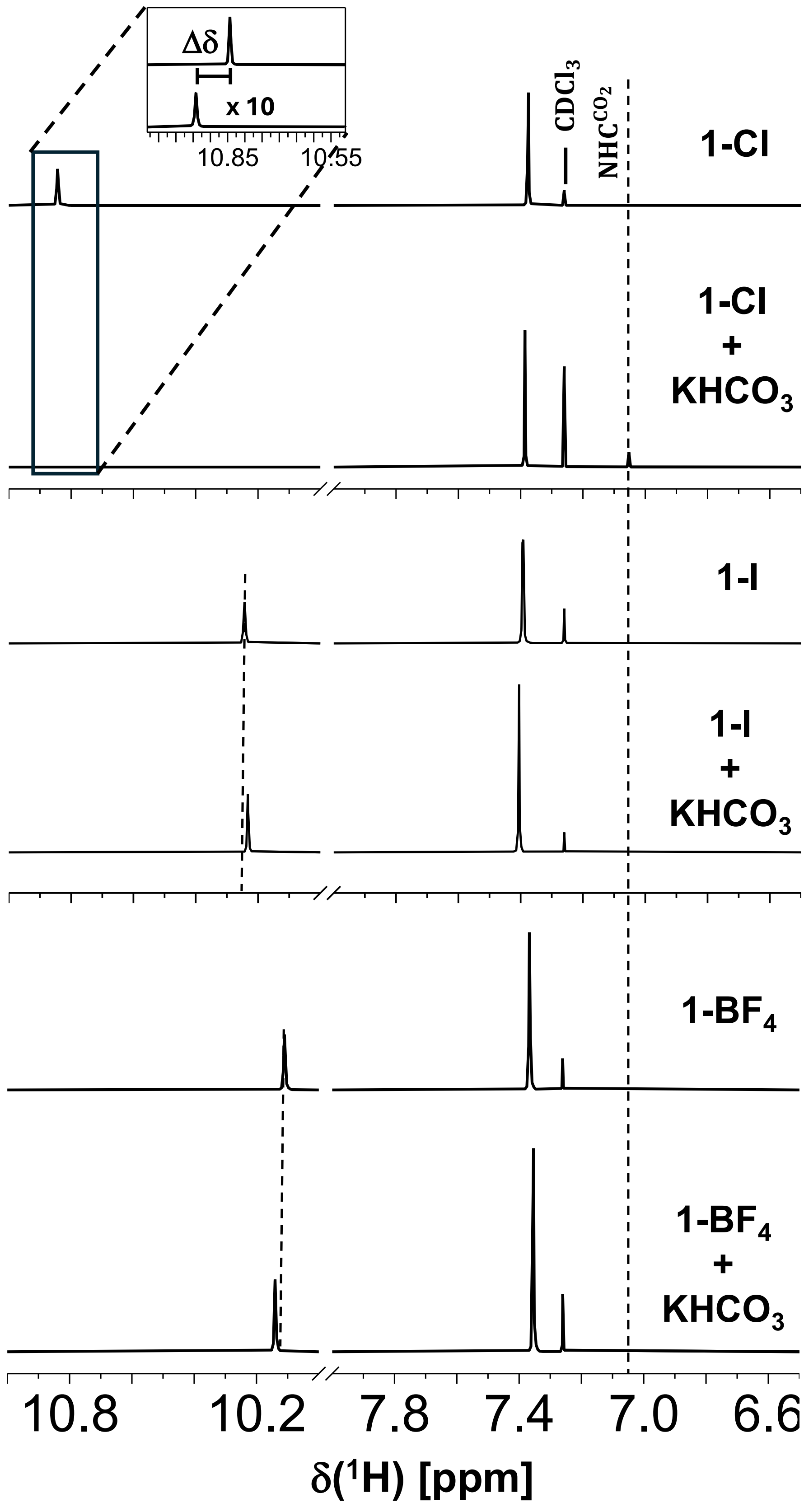

Figure S21. Comparison of ^{1}H-NMR spectra of the respective NHC precursors **1-Cl**, **1-I** and **1-BF$_4$** before and after treatment with $KHCO_3$ at room temperature in 1,4-dioxane for 24 h.

High-resolution mass spectrometry of an isolated intermediate in optimized reaction conditions

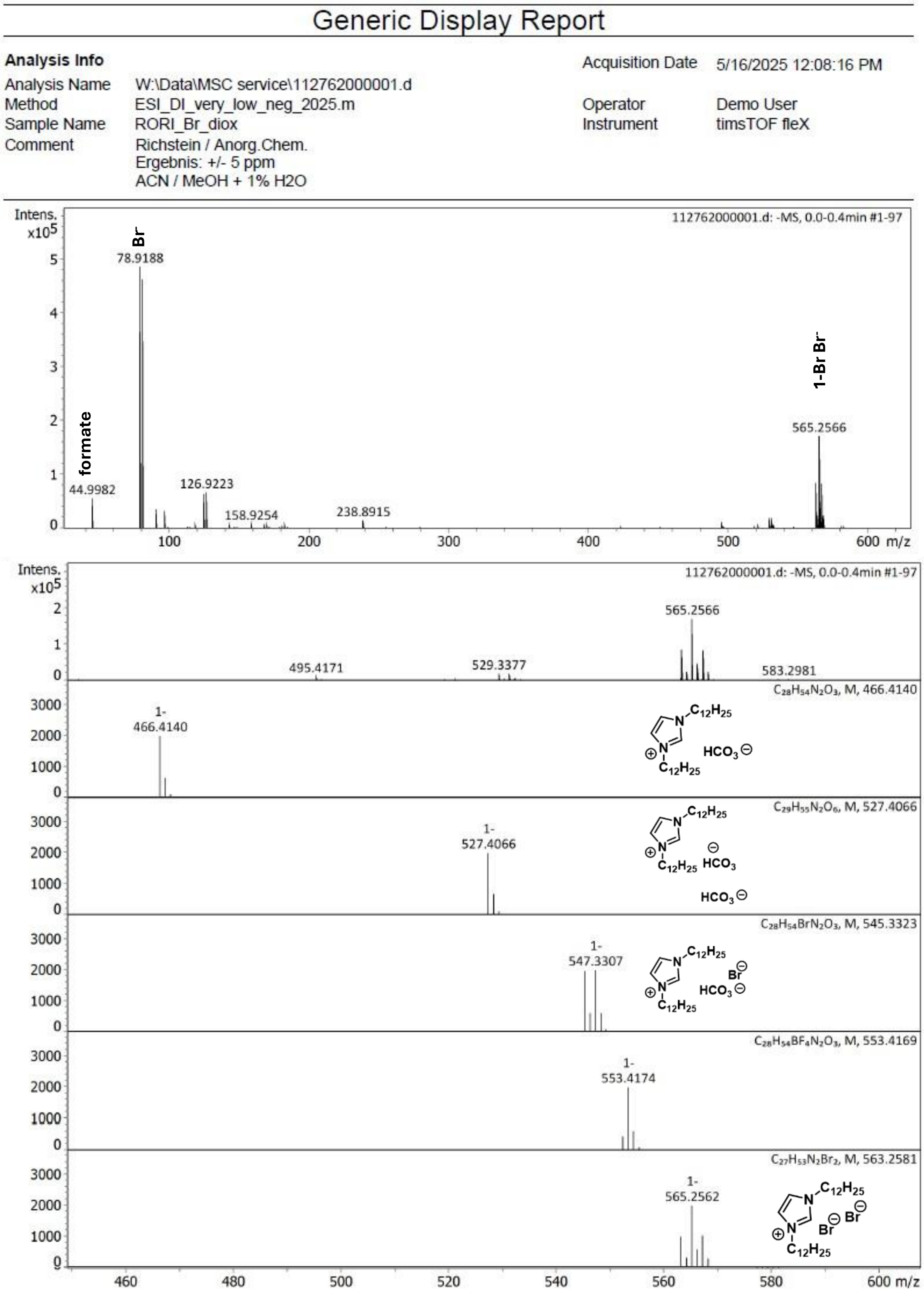

Figure S22. High resolution mass spectrometry of 1-Br after 24 h in 1,4-dioxane with 1.1 eq. $KHCO_3$. Surprisingly, neither potassium hydrocarbonate nor a respective adduct could be observed.

Full ^{1}H and ^{13}C NMR spectra of 4-Br

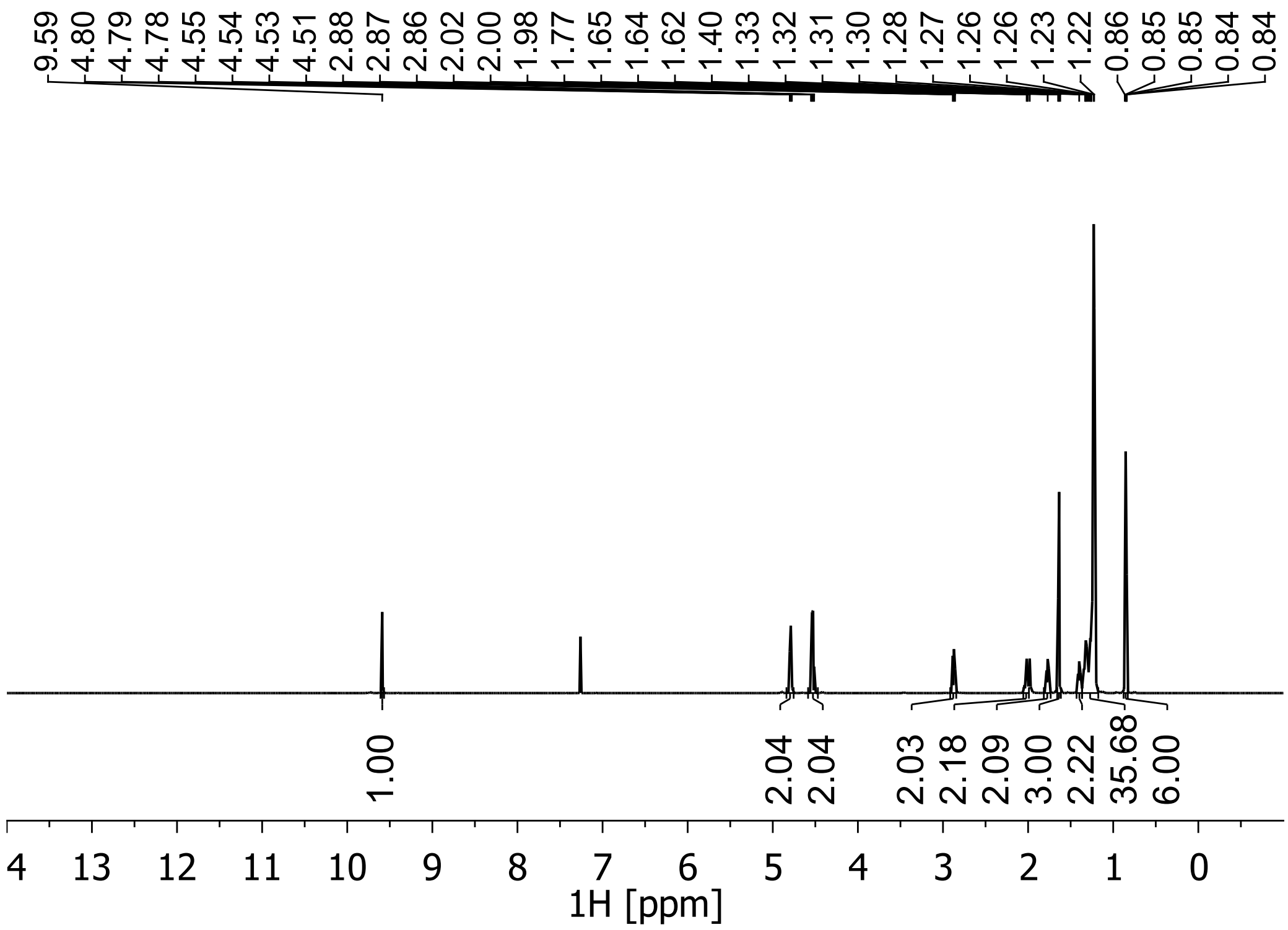

Figure S23. ^{1}H-NMR spectrum of **4-Br** in $CDCl_3$.

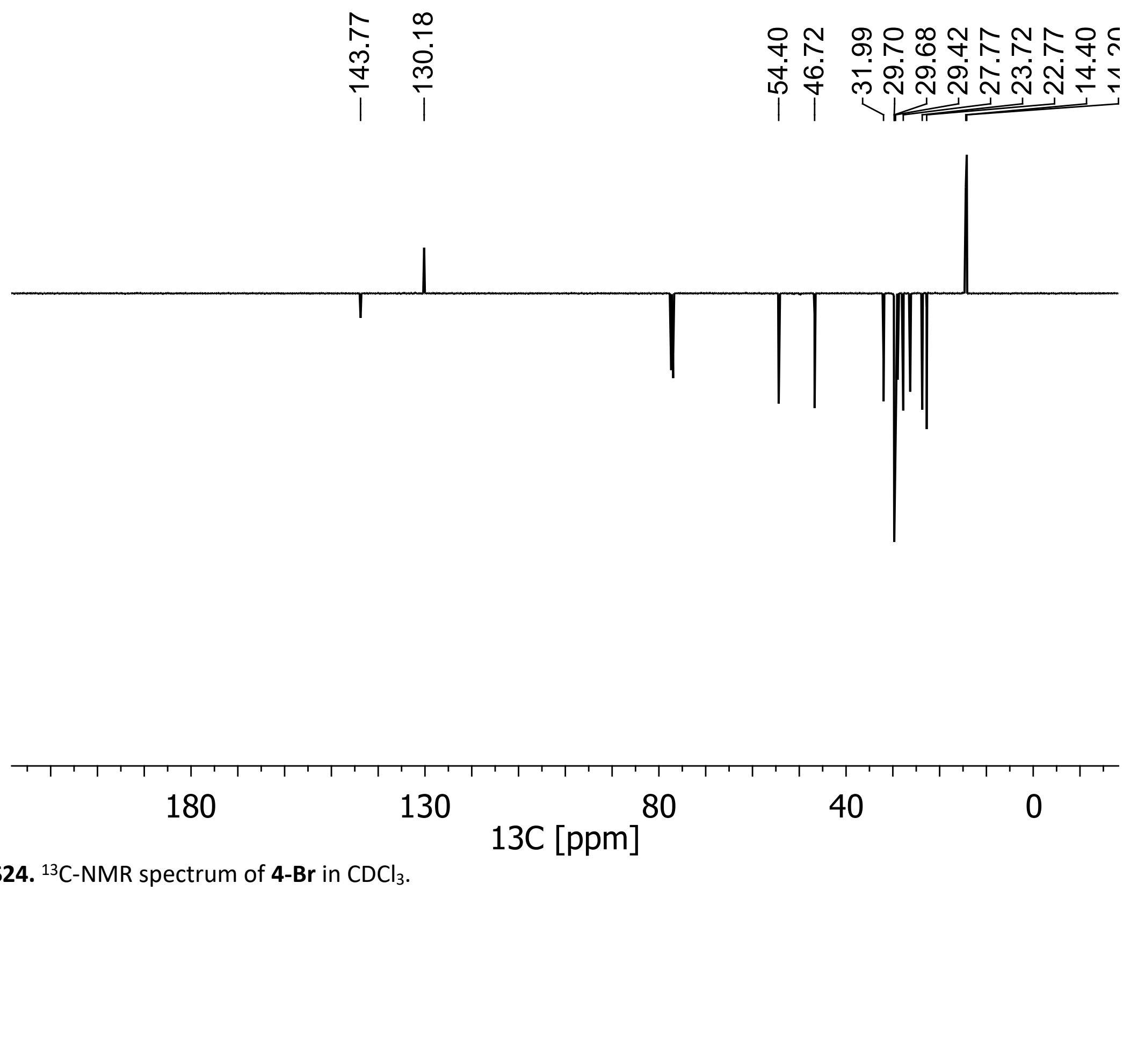

Figure S24. ^{13}C-NMR spectrum of **4-Br** in $CDCl_3$.

Supporting Literature

[1] K. Mebrouk, F. Camerel, O. Jeannin, B. Heinrich, B. Donnio, M. Fourmigué, *Inorganic Chemistry* **2016**, *55*, 1296-1303.

[2] H. V. Huynh, T. T. Lam, H. T. T. Luong, *RSC Adv* **2018**, *8*, 34960-34966.

[3] G. Damilano, D. Kalebić, K. Binnemans, W. Dehaen, *RSC Advances* **2020**, *10*, 21071-21081.

[4] N. Bridonneau, L. Hippolyte, D. Mercier, D. Portehault, M. Desage-El Murr, P. Marcus, L. Fensterbank, C. Chaneac, F. Ribot, *Dalton Trans* **2018**, *47*, 6850-6859.

[5] H. Zhao, F. W. Foss, R. Breslow, *Journal of the American Chemical Society* **2008**, *130*, 12590-12591.

[6] D. Majhi, J. Dai, S. V. Dvinskikh, *Physical Chemistry Chemical Physics* **2022**, *24*, 23532-23539.

[7] M. Lee, Z. Niu, C. Slebodnick, H. W. Gibson, *The Journal of Physical Chemistry B* **2010**, *114*, 7312-7319.

[8] V. Seidl, M. Sternberg, F. W. Heinemann, M. Schmiele, T. Unruh, P. Wasserscheid, K. Meyer, *Crystal Growth & Design* **2021**, *21*, 6276-6288.

[9] P. Małecki, K. Gajda, O. Ablialimov, M. Malińska, R. Gajda, K. Woźniak, A. Kajetanowicz, K. Grela, *Organometallics* **2017**, *36*, 2153-2166.

[10] M. Rivara, M. K. Patel, L. Amori, V. Zuliani, *Bioorganic & Medicinal Chemistry Letters* **2012**, *22*, 6401-6404.

[11] A. Porcheron, O. Sadek, S. B. Sowid, N. Bridonneau, L. Hippolyte, D. Mercier, P. Marcus, L. Mutalliev, C. Chauvier, C. Chanéac, L. Fensterbank, F. Ribot, *Chem. Mater.* **2023**, *35*, 6865-6876.

[12] Q. Liang, K. Hayashi, Y. Zeng, J. L. Jimenez-Santiago, D. Song, *Chem Commun (Camb)* **2021**, *57*, 6137-6140.

[13] E. C. Hurst, K. Wilson, I. J. S. Fairlamb, V. Chechik, *New J. Chem.* **2009**, *33*.

[14] J. D. Padmos, P. Zhang, *The Journal of Physical Chemistry C* **2012**, *116*, 23094-23101.

[15] E. C. Hurst, K. Wilson, I. J. S. Fairlamb, V. Chechik, *New Journal of Chemistry* **2009**, *33*.
